# A three-dimensional intestinal tissue model reveals factors and small regulatory RNAs important for colonization with *Campylobacter jejuni*

**DOI:** 10.1371/journal.ppat.1008304

**Published:** 2020-02-18

**Authors:** Mona Alzheimer, Sarah L. Svensson, Fabian König, Matthias Schweinlin, Marco Metzger, Heike Walles, Cynthia M. Sharma

**Affiliations:** 1 Chair of Molecular Infection Biology II, Institute of Molecular Infection Biology, University of Würzburg, Würzburg, Germany; 2 Department of Tissue Engineering and Regenerative Medicine, University Hospital Würzburg, Würzburg, Germany; 3 Fraunhofer-Institute for Silicate Research, Translational Centre Regenerative Therapies, Würzburg, Germany; 4 Core Facility Tissue Engineering, Otto-von-Guericke University, Magdeburg, Germany; The University of British Columbia, CANADA

## Abstract

The Gram-negative Epsilonproteobacterium *Campylobacter jejuni* is currently the most prevalent bacterial foodborne pathogen. Like for many other human pathogens, infection studies with *C*. *jejuni* mainly employ artificial animal or cell culture models that can be limited in their ability to reflect the *in-vivo* environment within the human host. Here, we report the development and application of a human three-dimensional (3D) infection model based on tissue engineering to study host-pathogen interactions. Our intestinal 3D tissue model is built on a decellularized extracellular matrix scaffold, which is reseeded with human Caco-2 cells. Dynamic culture conditions enable the formation of a polarized mucosal epithelial barrier reminiscent of the 3D microarchitecture of the human small intestine. Infection with *C*. *jejuni* demonstrates that the 3D tissue model can reveal isolate-dependent colonization and barrier disruption phenotypes accompanied by perturbed localization of cell-cell junctions. Pathogenesis-related phenotypes of *C*. *jejuni* mutant strains in the 3D model deviated from those obtained with 2D-monolayers, but recapitulated phenotypes previously observed in animal models. Moreover, we demonstrate the involvement of a small regulatory RNA pair, CJnc180/190, during infections and observe different phenotypes of CJnc180/190 mutant strains in 2D vs. 3D infection models. Hereby, the CJnc190 sRNA exerts its pathogenic influence, at least in part, via repression of PtmG, which is involved in flagellin modification. Our results suggest that the Caco-2 cell-based 3D tissue model is a valuable and biologically relevant tool between *in-vitro* and *in-vivo* infection models to study virulence of *C*. *jejuni* and other gastrointestinal pathogens.

## Author summary

Enteric pathogens have evolved numerous strategies to successfully colonize and persist in the human gastrointestinal tract. However, especially for the research of virulence mechanisms of human pathogens, often only limited infection models are available. Here, we have applied and further advanced a tissue-engineered human intestinal tissue model based on an extracellular matrix scaffold reseeded with human cells that can faithfully mimic pathogenesis-determining processes of the zoonotic pathogen *Campylobacter jejuni*. Our three-dimensional (3D) intestinal infection model allows for the assessment of epithelial barrier function during infection as well as for the quantification of bacterial adherence, internalization, and transmigration. Investigation of *C*. *jejuni* mutant strains in our 3D tissue model revealed isolate-specific infection phenotypes, *in-vivo* relevant infection outcomes, and uncovered the involvement of a small RNA pair during *C*. *jejuni* pathogenesis. Overall, our results demonstrate the power of tissue-engineered models for studying host-pathogen interactions, and our model will also be helpful to investigate other gastrointestinal pathogens.

## Introduction

Bacterial pathogens use a variety of virulence factors to colonize their host and to cause disease. For many pathogens, there is still much to be learned about the underlying molecular mechanisms of these processes, especially within the human host. While animal models and *in-vitro* two-dimensional (2D) cell culture systems have greatly contributed to our understanding of host-pathogen interactions, they can be limited in their ability to reflect certain aspects of disease development in the human host [1]. For example, epithelial 2D-monolayer cell cultures often do not recapitulate the complexity of native three-dimensional (3D) tissue [2]. They lack the interaction of eukaryotic cells with a fully developed 3D structure composed of extracellular matrix (ECM) proteins, and 2D cell monolayers generally do not achieve the 3D architecture of native parental epithelial tissue [3–6]. Moreover, cells grown in flat culture dishes often lack biochemically distinct polarity and in turn cannot respond to chemical and molecular gradients in three dimensions (apical, basal, and lateral) [5]. To partially overcome this, some cell lines can be cultured on porous 2D-Transwell (TW) inserts to facilitate development of apical-basolateral cell polarity and the formation of cell-cell junctions [7]. Nevertheless, they still lack the 3D architecture of *in-vivo* tissue. Thus, there is a need for advanced *in-vitro* cell culture models that can mimic complex characteristics of *in-vivo* tissue to study interactions of the mucosal epithelium with pathogens.

The Gram-negative Epsilonproteobacterium *Campylobacter jejuni* is currently the most prevalent cause of bacterial foodborne disease worldwide [8,9]. While *C*. *jejuni* is a commensal in avian species and livestock, it can cause severe gastroenteritis in humans by colonizing the intestinal epithelium and has also been linked to secondary neuropathies such as the Guillain-Barré syndrome (GBS) [10]. So far, little is known about what determines *C*. *jejuni* pathogenicity, and its genome sequence lacks homologs of dedicated virulence factors known from other enteric bacteria [11–13]. Many *C*. *jejuni* genes necessary for the interaction with host cells are related to motility [10,14], the flagellar filament [15], and the flagellar type three secretion system (T3SS), which is needed to secrete host-modulating effector proteins [16]. In addition, cell shape [17–19], metabolism [20–22], and surface carbohydrates such as the capsular polysaccharide (CPS) and lipooligosaccharide (LOS) [10] influence the interaction of *C*. *jejuni* with host cells.

While *in-vivo* colonization of *C*. *jejuni* is often investigated using chick models [11], its commensal lifestyle in avian species might not reflect host-pathogen interactions in humans. Mouse models are often not susceptible to efficient *Campylobacter* colonization [23,24] or do not exhibit signs of inflammatory disease unless the microbiome is depleted with antibiotics or if mice with genetic lesions, for example in genes encoding IL-10 [25] or the Single IgG IL-1 Related Receptor (SIGIRR) [26], are used. Interactions of *C*. *jejuni* with host cells are most commonly studied using cell lines seeded as *in-vitro* 2D-monolayers in multi-well dishes, while paracellular and transcellular migration as well as loss of barrier function during infection have been studied utilizing 2D-Transwell inserts [27,28]. However, as *C*. *jejuni* thrives mostly extracellularly, its natural human host environment cannot be easily recapitulated *in vitro* without the presence of an extracellular matrix, polarized expression of cell surface molecules, a 3D tissue architecture, and a mucosal epithelial barrier.

Here, we have developed and employed a dynamically cultured intestinal *in-vitro* 3D infection model based on tissue engineering technology to study pathogenesis of *C*. *jejuni*. Our model combines polarized Caco-2 cells with the 3D architecture provided by an extracellular matrix scaffold. It reveals strain-dependent virulence phenotypes of *C*. *jejuni* with respect to epithelial colonization and barrier disruption, and successfully recapitulates previously observed phenotypes of known *C*. *jejuni* colonization factors, such as its capsule or motility. Notably, the model reveals phenotypes for *C*. *jejuni* mutant strains that have previously only been detected in *in-vivo* animal models [29,30]. Moreover, we also uncovered a role for a bacterial small regulatory RNA (sRNA) pair in *C*. *jejuni* host interactions, which was surprisingly opposite in the 3D tissue model compared to 2D-monolayer infections. Overall, our intestinal 3D model can complement 2D cell culture and animal infection models and successfully reveal novel features of pathogen biology.

## Results

### Development of a dynamically cultured Caco-2 cell-based 3D small intestinal tissue model

**Fig 1:**
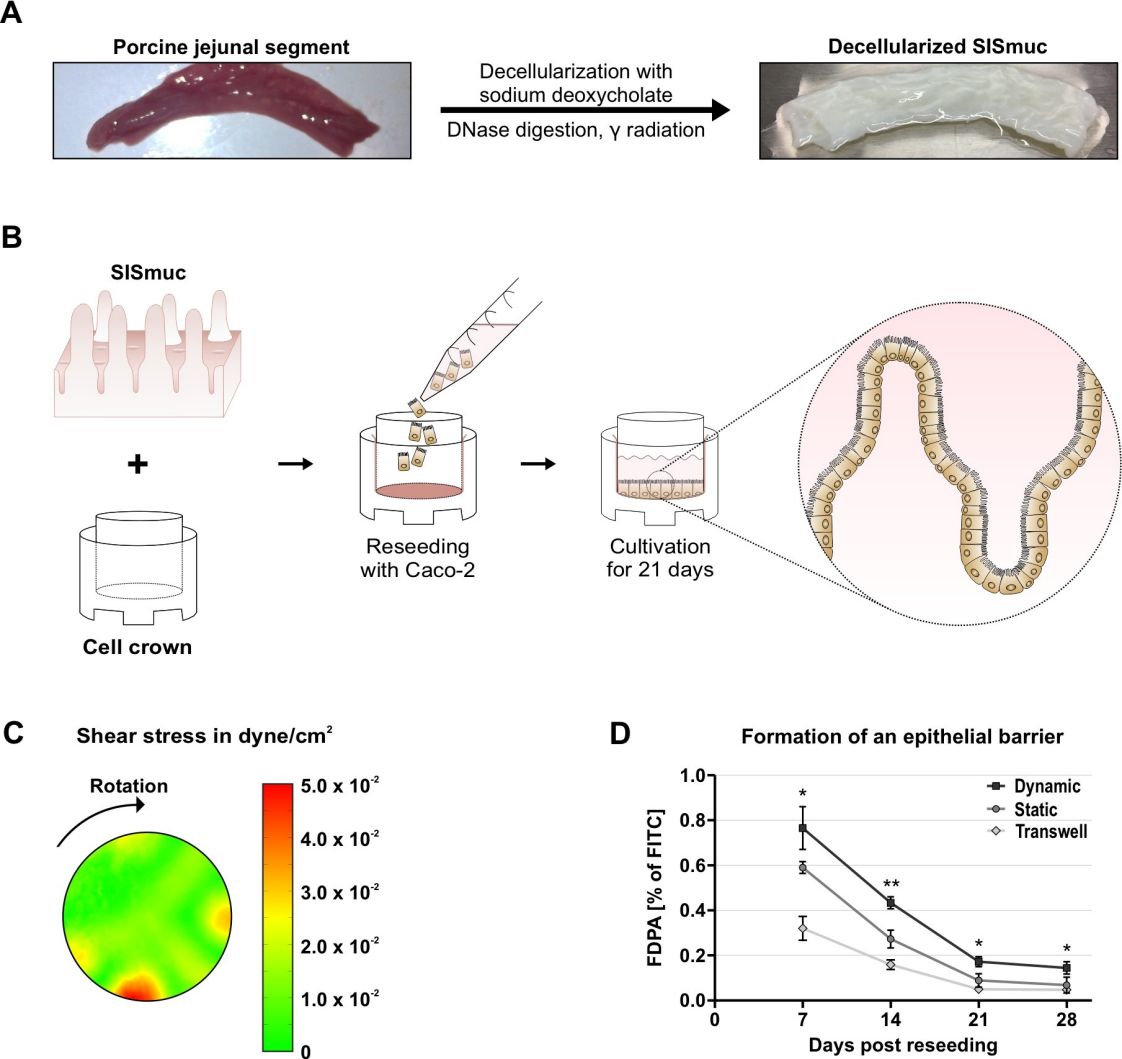
Set-up of a dynamically cultured Caco-2 cell-based 3D small intestine model. **(A)** The ECM SISmuc scaffold is generated by decellularization of a porcine jejunal segment with sodium deoxycholate, DNase digestion, and gamma radiation. **(B)** The SISmuc is fixed in between two metal rings (cell crown). The former mucosal side faces the apical compartment and is reseeded with Caco-2 cells and cultured for up to 28 days either statically or dynamically. **(C)** Simulation of fluid dynamics over the surface of a cell crown during orbital shaking with 65 rpm (clockwise rotation). **(D)** FITC-dextran permeability assays (FDPA) of Caco-2 cells reseeded on 2D-Transwells (0.4 μm pore size) or on SISmuc over 28 days of culture under static and dynamic conditions. FDPA values represent the percentage of input FITC-dextran that has diffused to the basolateral compartment. Error bars indicate standard deviations (SDs) of three independent biological replicates. Statistical analysis is indicated for permeability differences between the statically and dynamically cultured tissue models. **: *p* < 0.01, *: *p* < 0.05, using Student’s *t*-test.

Among 3D cell culture approaches [31,32], tissue engineering combines the support of scaffolds with the appropriate mammalian cell type to create *in-vitro* engineered complex 3D tissue models harboring the microarchitecture of human organs [33]. To develop such an infection model for *C*. *jejuni* infection studies, we built on a previously established tissue-engineered model for the small intestine, in which human Caco-2 cells are reseeded on the extracellular matrix scaffold SISmuc (Small Intestinal Submucosa) [34–36]. This scaffold is based on the so-called BioVaSc (Biological Vascularised Scaffold), which is generated by chemical decellularization of a porcine jejunal segment, leaving a structured ECM scaffold with its preserved native microarchitecture (Fig 1A) [34–36]. The SISmuc is then fixed into a cell crown and the former mucosal side is apically reseeded with Caco-2 cells (Fig 1B). Subsequent culture conditions can either be static in a cell culture incubator or under perfusion in a bioreactor system to provide mechanical stimuli to promote tissue differentiation [36] (S1A Fig). While Caco-2 cells cultured on SISmuc under static conditions already express intestinal mucosal cell markers such as the cell-cell junction proteins ZO-1 (zonula occludens-1) (S1B Fig), occludin, or E-cadherin, perfusion culture in a bioreactor leads to an improved 3D architecture, which more closely resembles the structure of human intestine (S1B Fig) [36]. However, as such perfusion bioreactors can only be set up for small scale experiments, we aimed to develop a dynamically cultured model that is easy to establish and increases the throughput capacity of these models to study infection processes.

To mimic microfluidic shear on the cells, we cultured the SISmuc reseeded with Caco-2 cells for up to 28 days on an orbital shaker inside a cell culture incubator. Using computational modeling, we predicted that a rotatory frequency of 65 rpm (rounds per minute) provides an average shear stress of 1.6 × 10^−2^ ±  4.7 × 10^−3^ dyne/cm^2^ across the scaffold surface for 12-well cell crowns (Fig 1C), mimicking mechanical stimulation measured for human intestine *in vivo* [37]. The modeling further predicted that the distribution of shear stress across the surface of a cell crown is fairly homogenous with hotspots of stronger shear stress around the periphery caused by the start and stop rotary movements of the shaker (Fig 1C). First, we investigated the epithelial barrier formation of the 3D model cultured at 65 rpm using an FITC-dextran permeability assay (FDPA) [38]. In comparison, permeability was also assessed for statically cultivated 3D tissue models and Caco-2 cells grown on 2D-Transwell inserts, which corresponds to polarized epithelial cells without the support of the SISmuc scaffold. While 2D-Transwells reached FDPA values of < 0.2% after 14 days, both statically and dynamically cultured 3D tissue models showed a delayed onset of epithelial barrier formation, indicated by higher permeability at days 7 and 14 (Fig 1D). This delay was even more pronounced for tissue models cultured under shear stress. While Caco-2 cells on 2D-Transwell inserts and the static SISmuc model reached similar FDPA values at later time points (21 and 28 days), the permeability of the dynamic model continued to be significantly higher. The tight barrier formation on 2D-Transwells is in line with previously reported higher transepithelial resistance (TER) values measured for Caco-2 cells on 2D-Transwell inserts compared to TER values of native human small intestine [39,40]. These results suggest that the epithelial barrier formed by Caco-2 cells under shear stress is less tight compared to either static culture on SISmuc or on 2D-Transwell inserts. Because all cell culture models did not show any further significant decrease of permeability after 21 days in culture and FDPA values remained stably low at 28 days post reseeding, all further downstream analyses were performed after 21 days in culture.

### Mechanical stimulation during dynamic culture positively influences tissue architecture and cell morphology

**Fig 2:**
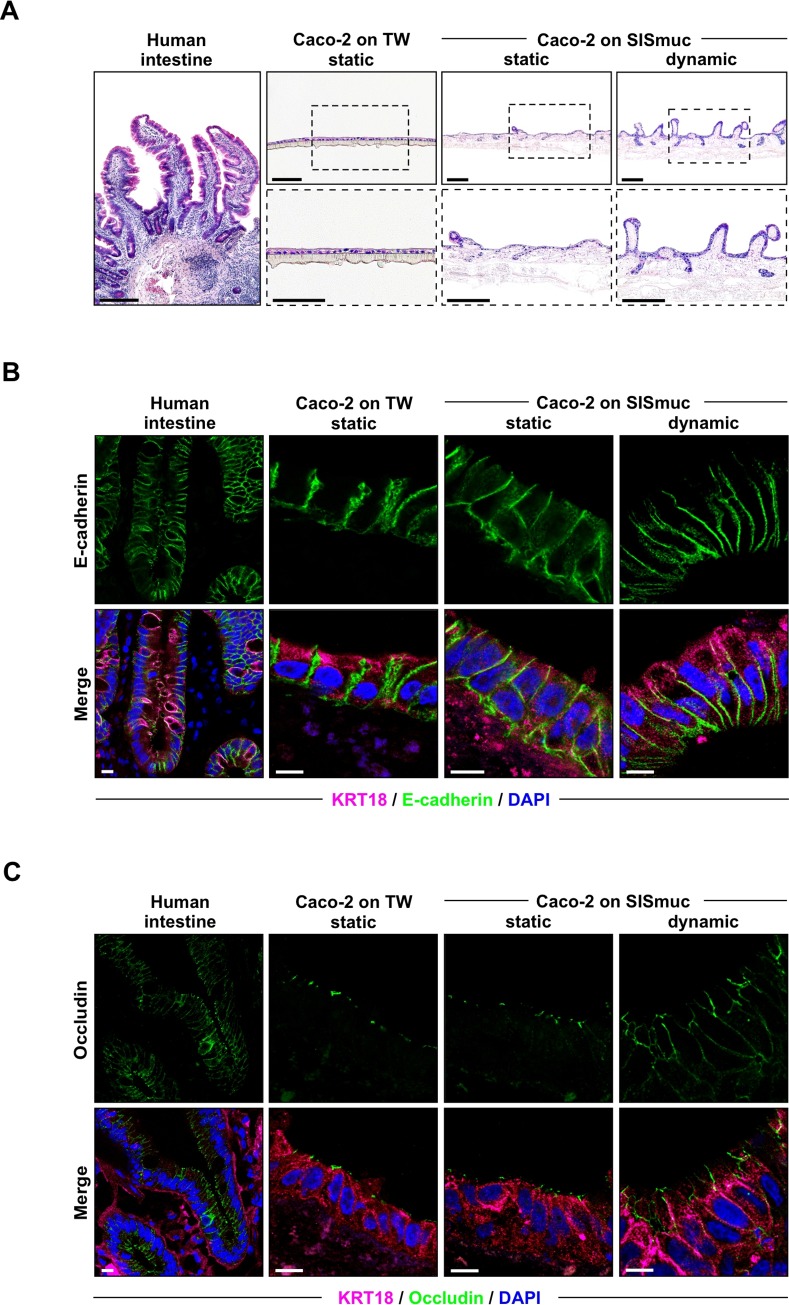
Dynamic culture enhances architecture and localization of cell-cell junction proteins of the Caco-2 tissue model. **(A)** Hematoxylin and Eosin (H&E) staining of paraffin sections of human small intestinal tissue (*left panel*) or Caco-2 cells on either 2D-Transwell (TW) inserts (*middle panel*) or SISmuc after 21 days in static or dynamic culture (*right panel*). Images below are higher magnifications of the center micrographs. Scale bars: 200 μm. **(B, C)** Confocal microscopy images of human small intestine (*left panel*), Caco-2 cells on TW (*middle panel*), as well as statically or dynamically cultured 3D tissue models (*right panel*). Tissues were stained with DAPI (nuclei, blue) and antibodies against KRT18 (cytokeratin 18, magenta), E-cadherin (AJ, green) **(B)**, and occludin (TJ, green) **(C)**. Scale bars: 10 μm.

To determine if the measured differences in paracellular permeability are accompanied by distinctive intestinal tissue characteristics, we performed microscopic analysis of cell morphology and three-dimensional mucosal architecture. Hematoxylin and Eosin (H&E) staining showed a confluent Caco-2 monolayer atop the respective support scaffolds (2D-Transwell or SISmuc), independent of their culture status (Fig 2A). Caco-2 cells on the 2D-Transwell exhibited a flat and unstructured organization. In contrast, the same cells cultured on the SISmuc showed 3D architectures resembling crypt- and villi-like structures. While this effect was only rarely visible under static conditions, dynamic culture seemed to improve tissue morphology and exhibited more pronounced villi and crypts reminiscent of a native human small intestine sample, which was stained in comparison.

Immunofluorescence staining of the adherens junction (AJ) protein E-cadherin (Fig 2B) and the tight junction (TJ) protein occludin (Fig 2C) showed the formation of more defined and properly localized cell-cell junctions at the periphery of the cells in the dynamically cultured tissue model compared to Caco-2 cells on either 2D-Transwell inserts or on SISmuc under static culture. Distinct apical localization of TJs could be detected in the dynamically cultured Caco-2 cell-based tissue model. Moreover, cells on 2D-Transwell inserts as well as in the static tissue model exhibited a more flattened cell shape, whereas cells cultured under fluidic shear exhibited an elongated structure more reminiscent of the highly prismatic morphology of mucosal enterocytes. As a quantitative readout for this observation, we measured the average height of cells cultured on the different scaffolds and during static and dynamic culture (S2 Fig). With increasing complexity of cell culture models (from static 2D-Transwell to static 3D tissue model to dynamic 3D tissue model) up to human biopsy samples, epithelial cells grew in height and reached columnar dimensions. Dynamic culture increased cell height about two- to three-fold when compared to Caco-2 cells on either SISmuc (static) or 2D-Transwell, respectively. Thus, epithelial cell morphology more closely resembled that of columnar enterocytes and with an average height of 21.52 ± 0.3125 μm reached nearly similar dimensions as measured for native human intestine and as previously reported [37,41].

Together with the measured barrier permeability that was closer to that of native tissue, the above microscopy results indicate that culture under dynamic conditions can simulate shear stress reminiscent of mechanical forces experienced *in vivo*. In turn, this led to a more pronounced microarchitecture suggestive of human intestine as well as to a stronger differentiation of the Caco-2 cells into a columnar mucosal barrier.

### *C*. *jejuni* can colonize and replicate on the 3D small intestinal tissue model

To test if we can use our 3D Caco-2 tissue model for infection studies with bacterial pathogens, we set up infection conditions in our 3D model for *C*. *jejuni*. *C*. *jejuni* isolates can show strong differences in gene repertoire and pathogenicity [42], and virulence of strains strongly correlates with their ability to adhere to and be internalized into host cells or to traverse polarized epithelial cell monolayers *in vitro* [43,44]. Thus, we compared two frequently studied *C*. *jejuni* strains, NCTC11168 and the more virulent 81–176. To calculate the number of bacteria required for a given multiplicity of infection (MOI), we first determined the mean final number of epithelial cells on each crown after 21 days of culture. Cell counting upon trypsin treatment and extensive mechanical dissolution (S1 Table) as well as determination of cell numbers based on DNA content using Pico-Green assays (S2 Table) showed that following static culture, approximately 675,000 Caco-2 cells are present on each cell crown. Similar average cell numbers where observed under dynamic conditions (S3 Table). This value was used to calculate an MOI of 20 for subsequent *C*. *jejuni* infection experiments.

**Fig 3:**
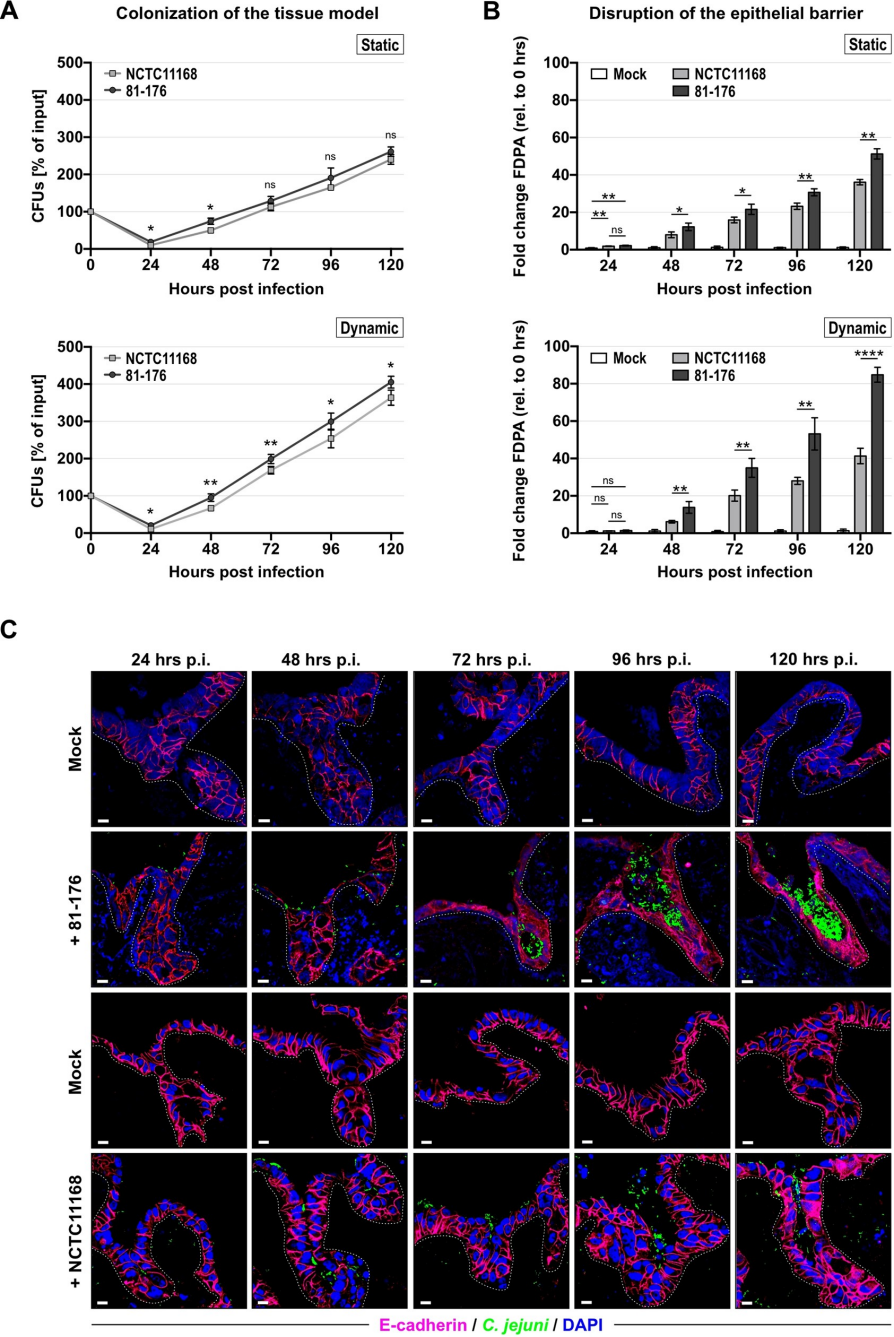
The 3D tissue model is colonized and disrupted by *C*. *jejuni* in a strain-specific manner. **(A)** CFU quantification (as percent of input) of *C*. *jejuni* strains NCTC11168 and 81–176 from 24–120 hours p.i. in the static (*upper panel*) and the dynamic (*lower panel*) 3D tissue model. Error bars indicate SDs of four biological replicates. **(B)** FDPA-based measurements of epithelial barrier disruption during *C*. *jejuni* infections of 3D tissue models cultured statically (*upper panel*) or dynamically (*lower panel*). Depicted is the mean of four independent experiments with corresponding SDs. Mock indicates non-infected controls. FDPA values are depicted as fold changes relative to the value at time point zero. Based on these fold changes, statistical significance was calculated between the two wild-type strains for each time point, as well as between NCTC11168/81-176 and the non-infected control at 24 hrs p.i. ****: *p* < 0.0001, **: *p* < 0.01, *: *p* < 0.05, ns: not significant, using Student’s *t*-test. **(C)** Confocal microscopy images of paraffin sections of the 3D tissue model cultured dynamically during infection with *C*. *jejuni* strain 81–176 (*upper panel*) or NCTC11168 (*lower panel*) and respective non-infected controls. Bacteria were visualized with an anti-*C*. *jejuni* antibody (green), nuclei were stained with DAPI (blue), and AJs were imaged using an anti-E-cadherin antibody (adherens junctions, magenta). Scale bars: 10 μm.

We established various readouts for the infection process, such as I) the isolation of colony forming units (CFUs) for bacterial adherence (ADH = adherence + internalization), internalization only (INT), as well as transmigration, II) measurement of potential disruption of the epithelial barrier function during infection, and III) immunohistochemical staining (IHC) for tissue and host cell morphology (S3A Fig). Utilizing a tissue punch, CFUs from the infected tissue model were isolated in a standardized way from equal sized tissue pieces with two previously used detergents [45,46], namely saponin (S3B Fig, *left panel*) and Triton X-100 (S3B Fig, *right panel*). A concentration of 0.1% of either detergent was sufficient to recover *C*. *jejuni* CFUs from the cell crowns. We infected both the pre-cultured static and dynamic 3D model (Fig 3A) as well as the static 2D-Transwell system (S4A Fig) with the two different *C*. *jejuni* wild-type strains. All experiments were conducted without further dynamic cultivation after infection. At 24 hrs p.i. (post infection) in the statically cultured tissue model, approximately 10% and 19% adhered and internalized bacteria of the input were recovered for strain NCTC11168 and 81–176, respectively (Fig 3A, *upper panel*). In the dynamic tissue model, *C*. *jejuni* colonization levels reached approximately 12% (NCTC11168) and 20% (81–176) after 24 hrs of infection (Fig 3A, *lower panel*). This indicates that culture conditions of the tissue model might not severely affect initial ADH and/or INT of *C*. *jejuni*.

Initial colonization rates in the 2D-Transwell system (6% for NCTC11168 and 8% for 81–176, S4A Fig) were lower for both wild-type strains when compared to the tissue models, which could be a result of the larger surface area provided by the ECM scaffold. Lower colonization of NCTC11168 in all three cell culture models might reflect strain-specific differences in adherence to/colonization of epithelial cells, as previously observed [47–49]. Starting from 24 hrs p.i., an increasing number of CFUs could be recovered from either tissue model for both strains, indicating that *C*. *jejuni* survives and replicates on the 3D tissue models. While the lower number of recovered CFUs of strain NCTC11168 compared to 81–176 at 24 hrs p.i. was also observed until 120 hrs p.i. in the dynamically cultured tissue model (Fig 3A, *lower panel*), no significant difference between colonization by NCTC11168 or 81–176 could be observed in the static system or the 2D-Transwell model at 72–120 hrs p.i. (Fig 3A, *upper panel*, S4A Fig). The significantly attenuated colonization in the dynamic model observed for strain NCTC11168 compared to 81–176 (Fig 3A, *lower panel*) suggests that its microenvironment might be able to uncover more subtle differences in colonization phenotypes between *C*. *jejuni* strains also during longer infection times. Comparable CFUs of both strains in the supernatant of the respective cell culture models or in cell culture medium alone indicated that the observed colonization differences are not due to different bacterial growth of the two *C*. *jejuni* wild-type strains (S5A and S5B Fig). Overall, CFU levels of both wild-type strains at various time points post infection appeared to be higher in the dynamically cultured tissue model compared to its static counterpart or the 2D-Transwell, suggesting the presence of an environment more supportive of *C*. *jejuni* growth and/or survival. Thus, infection of the Caco-2 cell-based tissue models shows that *C*. *jejuni* can use it as a replicative niche.

### *C*. *jejuni* strains show variations in the disruption of the epithelial barrier

Next, we investigated whether colonization with *C*. *jejuni* leads to disruption of the epithelial barrier of the cell culture models. FDPA measurements during the time course of infection revealed that the presence of either strain results in an increasing loss of barrier integrity for both the static and the dynamic 3D tissue model as well as for the 2D-Transwell system (Fig 3B, S4B Fig). While no significant increase in permeability was observed in the dynamic system after 24 hrs p.i. (Fig 3B, *lower panel*), tissue models pre-cultured under static conditions and 2D-Transwells already showed an increase in permeability of two- to three-fold and eight- to nine-fold, respectively (Fig 3B, *upper panel*, S4B Fig). A delayed onset of increasing permeability in the dynamically cultured tissue model, even with comparable bacterial loads, hints towards greater resistance of pathogen-induced leakage of the epithelium.

The barrier function decreased in all infection models over time (up to 120 hrs p.i.). Despite lower colonization rates in the 2D-Transwell system, permeability measurements of infected 2D-Transwells indicated an overall stronger disruption of the epithelial barrier at all time points compared to the static and the dynamic tissue model (S4B Fig). This suggests a lower capability of the 2D-Transwell model to withstand *C*. *jejuni*-induced disruption of the epithelial barrier compared to the 3D tissue models. In addition, permeability increased to a greater extent in the dynamic tissue model compared to the static model (Fig 3B), indicating that this model might provide initial protection against *C*. *jejuni*-induced disruption of barrier function but overall does not prevent opening of epithelial cell-cell junctions. At early stages of the infection (24 and 48 hrs p.i.), permeability measurements revealed a higher disruption of the epithelial barrier by strain 81–176 compared to NCTC11168 in all tested cell culture models (Fig 3B, S4B Fig). While in the 3D tissue models permeability measurements continued to be significantly different between NCTC11168 and 81–176, this continued isolate-dependent disruption of epithelial barrier function could not be observed during infection of the 2D-Transwell. The strain-specific difference in opening of the epithelial barrier was most pronounced in the dynamically cultured 3D model (Fig 3B, *lower panel*) and might reflect the greater potential of strain 81–176 to colonize this particular tissue model compared to NCTC11168. Immunofluorescence staining of E-cadherin (Fig 3C) and occludin (S6 Fig) in the dynamically cultured tissue model infected with *C*. *jejuni* strain 81–176 compared to non-infected controls revealed redistribution of these two cell-cell junction proteins from the lateral membranes to an intracellular location. This was mostly visible during later stages of the infection (72–120 hrs p.i.). In particular, while only a small fraction of the AJ protein E-cadherin was diffusely localized at early stages of the infection process (24 and 48 hrs p.i.), the protein was mostly confined to the periphery of the cells compared to non-infected controls (Fig 3C, *upper panel*). In addition, a distinct apical localization of the TJ protein occludin could be observed in non-infected control crowns throughout the culture period (24–120 hrs p.i.) (S6 Fig). This pattern of apical localization was diminished upon infection with *C*. *jejuni* strain 81–176 and also resulted in an intracellular redistribution of occludin (S6 Fig, insets, white arrows). This observation was not restricted to areas of direct contact between epithelial cells and bacteria. When models were infected with strain NCTC11168, intracellular redistribution of the AJ protein E-cadherin (Fig 3C, *lower panel*) and the TJ protein occludin (S6 Fig) from their peripheral and apical locations, respectively, occurred later compared to those infected with 81–176. In addition, the pattern for both adherens and tight junctions appeared to be less disrupted for NCTC11168 when compared to tissue models infected with 81–176. This was particularly apparent in the case of AJs, where up to 96 hrs p.i. the distribution pattern of E-cadherin still appeared to be mostly confined to the periphery of the host cell in NCTC11168-infected tissue models (Fig 3C, *lower panel*). Throughout the infection process, bacterial cells of strain 81–176 accumulated in the crypts of the tissue model (Fig 3C, 72–120 hrs p.i.). This observation has previously been reported for *in-vivo* animal infection studies with *C*. *jejuni* [11,26,50] but, to the best of our knowledge, has not been recapitulated with *in-vitro* models so far. Even though strain NCTC11168 could also be observed close to or inside crypts, it did not appear to accumulate to such high numbers compared to 81–176 (Fig 3C, *lower panel*). Because the tissue model cultured under dynamic shear fluid conditions was not significantly disrupted during initial (24 hrs p.i.) *C*. *jejuni* infection and also showed greater sensitivity in distinguishing strain-specific colonization patterns, all subsequent infection experiments were performed with the dynamically cultured 3D model.

### Transmigration by *C*. *jejuni* is delayed in the 3D tissue model compared to 2D-Transwell inserts

**Fig 4:**
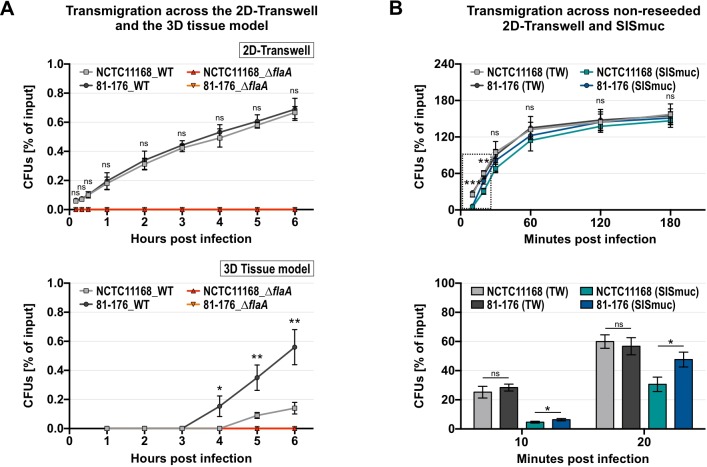
*C*. *jejuni* transmigration across the 3D tissue model is delayed compared to 2D-Transwell infections. **(A)** Caco-2 cells were grown statically on 2D-Transwells (pore size 3 μm) (*upper panel*) or dynamically on SISmuc scaffold (*lower panel*). Transmigration of *C*. *jejuni* wild-type strains 81–176 and NCTC11168 and their respective Δ*flaA* deletion mutant strains (Δ*flaA*) was determined by isolating CFUs up to six hours p.i. from the basolateral compartments. Experiments were conducted four times and CFUs are depicted as the percentage of input CFUs. Graphs represent the mean value with corresponding SDs, where significance was calculated for the recovered CFUs between the two wild-type strains at each time point. **(B)** CFUs from the basolateral compartment of non-reseeded 2D-Transwell (TW) and SISmuc from 10 min to 180 min p.i. (*upper panel*) with *C*. *jejuni* wild-type strains NCTC11168 and 81–176. For either wild-type strain, CFUs from the basolateral compartment of the 2D-Transwell were compared to those recovered from the basolateral compartment of the 3D tissue model. Asterisks at the early time points (10 min and 20 min p.i.) indicate a significant difference in transmigration between 2D-Transwell and 3D tissue model for both NCTC11168 and 81–176 (*upper panel*), as well as between NCTC11168 and 81–176 in the 3D tissue model only (*lower panel*). Early time points (10 min and 20 min p.i.) are depicted separately as a bar graph (*lower panel*) for better resolution. Experiments were conducted four times and graphs represent the mean value with corresponding SDs. ***: *p* < 0.001, **: *p* < 0.01, *: *p* < 0.05, ns: not significant, using Student’s *t*-test.

To reach deeper tissue regions, intestinal pathogens have to cross the epithelial barrier [27,51], which is also considered a major contributor to tissue damage elicited by *C*. *jejuni* [11]. To investigate whether *C*. *jejuni* can transmigrate through the epithelial barrier of the dynamically cultured 3D tissue model, we quantified the number of transmigrated bacteria of *C*. *jejuni* strains 81–176 and NCTC11168 and compared it to their transmigration behavior in the 2D-Transwell system (Fig 4A). Motility is required for *C*. *jejuni* colonization *in vivo* and *in vitro* [11,15,27], as well as for its transmigration through polarized epithelial cells [52]. Thus, as controls we also examined transmigration of isogenic, non-motile Δ*flaA* mutant strains, which lack the major flagellin FlaA that is required to build a WT flagellar filament. While increasing numbers of wild-type bacteria were detected in the basolateral compartment of the 3D model and 2D-Transwell system for both strains following inoculation, no CFUs could be recovered from the basolateral compartment for the Δ*flaA* mutant strains in either cell culture model (Fig 4A). Interestingly, both *C*. *jejuni* wild-type strains needed approximately eight times longer to cross the epithelial barrier provided by the 3D tissue model (Fig 4A, *lower panel*) than by the 2D-Transwell system (Fig 4A, *upper panel*). Although FDPA measurements suggested that the epithelial barrier of the uninfected 2D-Transwell is even less permeable than the 3D tissue model (Fig 1D), the bacteria were able to cross this barrier in less than 30 min. As the supporting scaffolds in these two systems differ (ECM vs. polycarbonate membrane), we also tested the migration behavior of both *C*. *jejuni* wild-type strains through non-reseeded SISmuc and 2D-Transwell (Fig 4B). Transmigration across the SISmuc scaffold could now already be observed after ten minutes. Whereas transmigration of both strains was significantly lower across the ECM scaffold compared to the 2D-Transwell at these early time points (10–20 min p.i.), this difference was no longer visible at later time points (30–180 min p.i.) (Fig 4B). This suggests that while the SISmuc scaffold poses a slightly greater obstacle for bacterial transmigration, it does not completely account for the time difference of three to four hours when each scaffold structure contains an additional epithelial barrier provided by the Caco-2 cells.

For the 2D-Transwell, both 81–176 and NCTC11168 crossed the epithelial barrier at comparable times (Fig 4A, *upper panel*). However, in the 3D tissue model, NCTC11168 reached the basolateral side almost one hour later than 81–176 (Fig 4A, *lower panel*). This could in part be due to a potential strain-specific interaction with extracellular matrix proteins, as NCTC11168 showed a significantly reduced number of transmigrated CFUs through an empty SISmuc scaffold when compared to 81–176 at early time points (Fig 4B, *lower panel*).

### Adherence and internalization by *C*. *jejuni* is delayed in 3D compared to 2D-monolayer infections

**Fig 5:**
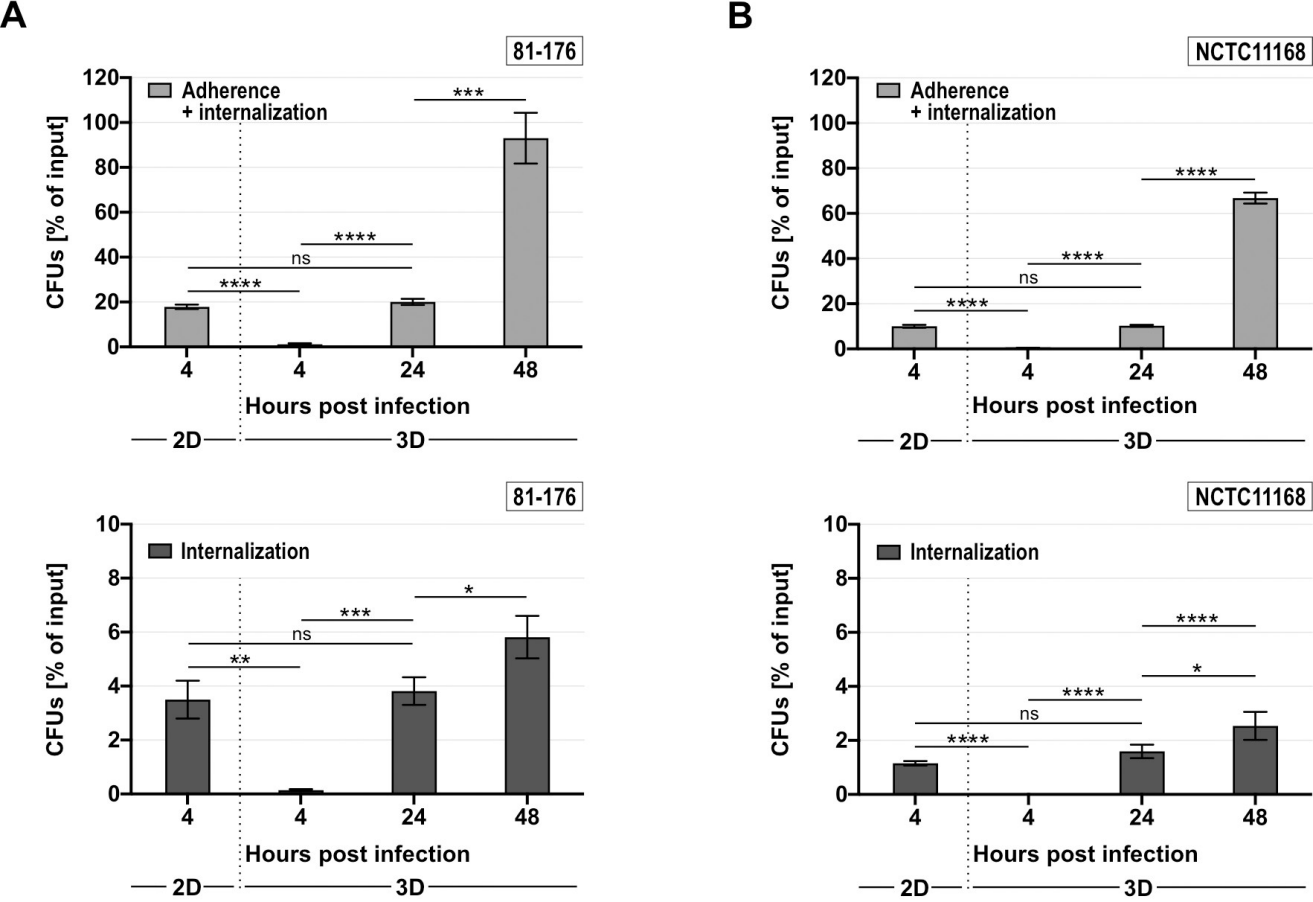
Adherence and internalization of *C*. *jejuni* is impeded in the 3D tissue versus 2D-monolayer environment. **(A, B)** Adherence (*upper panels*) and internalization (*lower panels*) of *C*. *jejuni* strains 81–176 **(A)** and NCTC11168 **(B)** were examined at four hrs p.i. in 2D-monolayers and 4–48 hrs p.i. in the 3D tissue model. Bars represent the mean of four independent experiments with respective SDs. ****: *p* < 0.0001, ***: *p* < 0.001, **: *p* < 0.01, *: *p* < 0.05, ns: not significant, using Student’s *t*-test.

To study adhesion and internalization of *C*. *jejuni* in our 3D model, we compared infections of the 3D tissue model with *C*. *jejuni* strains 81–176 and NCTC11168 to infections of non-polarized Caco-2 cells seeded as a 2D-monolayer in a 6-well dish system. At 4 hrs p.i. during 2D-monolayer infection, *C*. *jejuni* strain 81–176 (Fig 5A) reached ADH (*upper panel*) and INT (*lower panel*) rates of 17.9% and 2.6%, respectively, which were significantly higher compared to 10.1% and 1.2% observed for strain NCTC11168 (Fig 5B). In contrast, very few cell-associated bacteria could be isolated in our 3D tissue model for either wild-type strain at 4 hrs p.i. (Fig 5A and 5B, *upper panels*). In particular, internalization was almost undetectable at this early time point (Fig 5A and 5B, *lower panels*). Nevertheless, similar to what was observed for cell-associated bacteria (Fig 3A), the number of internalized bacteria increased up to 72 hrs p.i. for both *C*. *jejuni* wild-type strains but continued to be very low (S7A Fig). Internalization rates declined at later time points, indicating that internalized bacteria, especially during these later time points of the infection (96 and 120 hrs p.i.), contribute only a very low percentage to the overall increasing number of CFUs isolated from the tissue models over time (Fig 3A). Likewise, intracellular survival curves revealed that a significant number of CFUs, albeit strongly decreasing, could be isolated for both wild-type strains up to 72 hrs p.i. (S7B Fig). Strain 81–176 showed significantly higher levels of bacteria surviving intracellularly at each time point up to 96 hrs. These results suggest that also in the 3D tissue model, *C*. *jejuni* is not able to replicate intracellularly, which is in line with what has previously been observed with various gastrointestinal cell lines [27]. Thus, the increasing number of intracellular CFUs measured up to 72 hrs p.i. in the 3D tissue model most likely stems from extracellularly replicating bacteria, which are subsequently internalized into host cells.

Comparison of CFU percentages between 2D-monolayer and 3D cell culture model revealed that after 24 hrs p.i. in the 3D tissue model, ADH and INT rates reached levels equal to those observed in the 2D-monolayer infections at 4 hrs p.i., irrespective of the *C*. *jejuni* strain background (Fig 5A and 5B). Thus, the onset of infection seems to be delayed in the three-dimensional tissue environment compared to a two-dimensional non-polarized monolayer. Moreover, while our 3D tissue model enabled the assessment of infection over a longer time course, infection in the conventional 2D-monolayer system could not exceed 12 hrs due to increasing death of the host cells. While we did observe host cell death occurring in the 3D model mainly at later time points (96 and 120 hrs p.i.), the tissue models remained generally intact over the course of the experiment. Taken together, these data support the premise that the 3D tissue model poses a greater obstacle for *C*. *jejuni* to overcome than 2D-monolayer or 2D-Transwell systems in regards to adherence, internalization, and transmigration.

### The 3D tissue model reveals phenotypes of *C*. *jejuni* mutants that are not apparent in conventional 2D-monolayer assays

**Fig 6:**
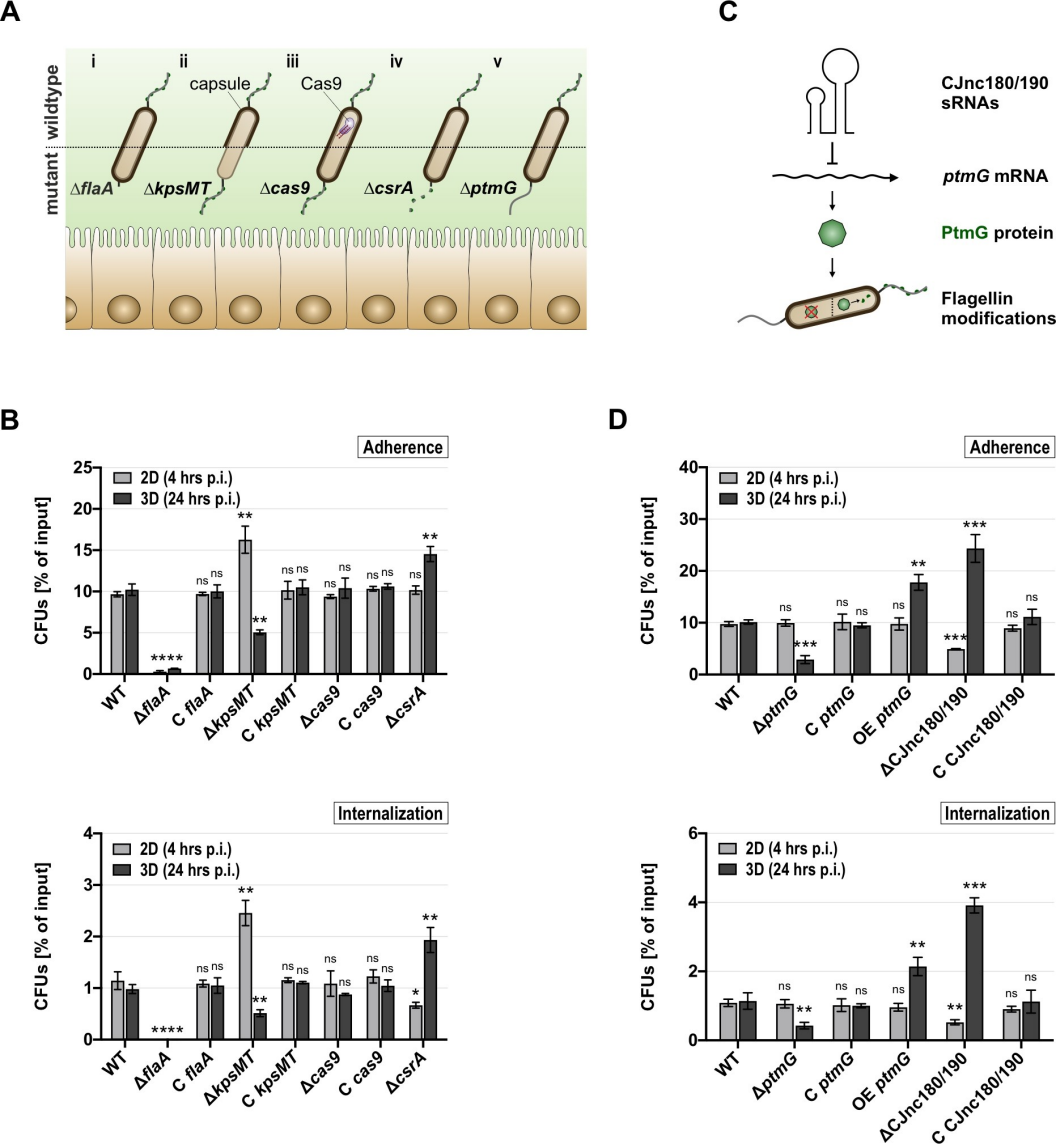
Infection with *C*. *jejuni* NCTC11168 deletion mutants differs between 2D-monolayer and 3D tissue model infections. **(Ai-v)** Schematic comparison between wild-type and respective phenotypic manifestation by deletion of *flaA* **(i)**, *kpsMT* **(ii)**, *cas9* **(iii)**, *csrA* **(iv)**, and *ptmG* **(v)**. **(C)** The sRNA pair CJnc180/190 post-transcriptionally represses *ptmG* mRNA, which in turn is involved in the legionaminic acid flagellin glycosylation pathway in *C*. *jejuni*. **(B, D)** Isolation of CFUs from 2D-monolayers (4 hrs p.i.) or tissue models (24 hrs p.i.) for *C*. *jejuni* NCTC11168 wildtype (WT), deletion mutants (Δ*flaA*, Δ*kpsMT*, Δ*cas9*, Δ*csrA*), and their respective complementation strains (C *flaA*, C *kpsMT*, C *cas9*) **(B)**, as well as for Δ*ptmG*, ΔCJnc180/190 in addition to their complementation (C *ptmG*, C CJnc180/190) and overexpression (OE *ptmG*) strains **(D)**. CFUs are depicted as the percentage of their respective input CFUs and represent the mean of three biological replicates with corresponding SDs. Statistical significance was calculated between wild-type CFUs (2D/3D) and those recovered for each mutant strain (2D/3D). Thus, asterisks or ns above each bar indicate the significance of the tested mutant compared to their respective wildtype in 2D or 3D. ****: *p* < 0.0001, ***: *p* < 0.001, **: *p* < 0.01, *: *p* < 0.05, ns: not significant, using Student’s *t*-test.

So far, our study suggested that the presence of the microarchitecture of the intestine in the 3D tissue model could influence host-pathogen interactions and reveal phenotypes in terms of *C*. *jejuni* adherence, colonization/replication, internalization, transmigration, as well as bacteria-induced barrier disruption. To test the involvement of diverse aspects of *C*. *jejuni* physiology during infection of our 3D tissue model, representative deletion mutants of strains 81–176 and NCTC11168 (Fig 6A) were evaluated for their colonization ability. The non-motile Δ*flaA* deletion mutant lacking a wild-type filament (Fig 6Ai and S8 Fig) was used to study the effects of motility, which has previously been associated with an inability to invade epithelial cells *in vitro* [15] as well as with a lack of animal colonization *in vivo* [26,53]. The polysaccharide capsule of *C*. *jejuni* has also been shown to influence virulence [26,54]. A double deletion mutant (Δ*kpsMT*) of the capsule ABC transporter and its corresponding ATP-binding protein lacks the capsule, while having no significant effect on bacterial swimming behavior in strain NCTC11168 (Fig 6Aii and S8A Fig) and only a minor decrease in motility in strain 81–176 (S8B Fig). Several *C*. *jejuni* isolates, including NCTC11168, possess a Type II-C CRISPR/Cas system [55]. Because there is emerging evidence that CRISPR/Cas systems could, beyond their function as prokaryotic immune systems, also affect virulence of pathogens, including *C*. *jejuni* [56], we included a motile Δ*cas9* mutant lacking the CRISPR/Cas Type II nuclease Cas9 in strain NCTC11168 (Fig 6Aiii and S8A Fig). As the CRISPR/Cas system is absent in the 81–176 wild-type isolate, this particular mutant was only tested in NCTC11168. Last, we examined a deletion mutant of the translational regulator CsrA (Fig 6Aiv), which has previously been implicated in *C*. *jejuni* biofilm formation, oxidative stress response, and infection [57]. It binds and regulates the *flaA* mRNA (encoding the major flagellin) as well as several other flagellar mRNAs, and impacts motility [58–60] (S8 Fig).

The above-described deletion mutants were tested in the 3D tissue model for their potential to adhere to and to get internalized into polarized intestinal epithelial cells in comparison to their ability to interact with a non-polarized 2D-monolayer of Caco-2 cells. We compared 4 hrs p.i. in the 2D-monolayer with 24 hrs p.i. in the dynamically cultured 3D model, which we had previously determined as the time points where a similar number of CFUs can be observed in the two different models (Fig 5). The Δ*flaA* mutant in both *C*. *jejuni* strain backgrounds showed almost no ADH to or INT into Caco-2 cells, neither in 2D nor in 3D (Fig 6B and S9A Fig). This phenotype had been observed previously in 2D cell lines [15] as well as in various animal models [26,53,61], suggesting that *C*. *jejuni* motility is equally important for host cell interaction in the 3D tissue model. While the *C*. *jejuni* strains lacking the capsule surrounding the bacterial cell (Δ*kpsMT*) showed increased ADH and INT in conventional 2D-monolayer infections, the opposite phenotype was detected in the 3D tissue model (Fig 6B and S9A Fig). Interaction of *kpsMT*-deficient bacteria with the reconstructed polarized intestinal epithelium yielded a significantly lower percentage of bacterial cells attached to or internalized into host cells when compared to its parental wild-type. The infection phenotypes observed for the Δ*flaA* and Δ*kpsMT* deletion mutants in both NCTC11168 and 81–176 could be fully complemented by expression of the respective genes from the unrelated *rdxA* locus (Fig 6B and S9A Fig). In line with previous observations [56], deletion of *cas9* in strain NCTC11168 had no significant effect on bacterial ADH and/or INT in a 2D or 3D cell culture environment (Fig 6B). This suggests that also in the 3D tissue model, Cas9 does not play an obvious role in influencing host-pathogen interactions for strain NCTC11168.

Deletion of the translational regulator CsrA in NCTC11168 showed no significant reduction in ADH when compared to the wildtype in 2D-monolayer infections (Fig 6B, *upper panel*). However, internalization into non-polarized Caco-2 cells was significantly diminished (Fig 6B, *lower panel*), indicating a dispensability of CsrA in this particular strain background for the initial adhesion to the host cell surface but a role during the internalization process and/or intracellular survival. In the 3D model, *csrA*-deficient NCTC11168 showed an increase in both ADH and INT (Fig 6B). While we were unable to generate a *csrA* complementation strain in NCTC11168, these seemingly opposite phenotypes of a Δ*csrA* mutant in this *C*. *jejuni* isolate in 2D-monolayer compared to 3D infection experiments suggest a distinctive function of this regulator and its impact on motility, as well as potentially different or additional targets during colonization and survival in these contrasting environments. Infection in 2D-monolayers with an 81–176 Δ*csrA* mutant showed the same trend as previously reported for this strain with non-polarized host cells (decreased ADH, increased INT) (S9A Fig) [57]. In the 3D tissue model, the number of cell-associated bacteria (ADH) for this deletion mutant was also decreased compared to wildtype and a complementation strain in 81–176 (S9A Fig, *upper panel*). In contrast to an increased recovery of intracellular CFUs from 2D-monolayers, internalization levels in the 3D tissue models were decreased for the mutant strain (S9A Fig, *lower panel*). This suggests that *csrA* and its targetome in the two *C*. *jejuni* isolates impacts the infection process differently depending on either the 2D or 3D environment faced by the bacteria. To account for the possibility that a different growth phase of the *C*. *jejuni* mutant strains after 24 hrs in the tissue model has an impact on the infection process, we also tested all deletion and complementation strains in the 3D tissue model after 4 hrs of infection. Although the CFU recovery rate at this early time point is very low, we observed the same trend for all strains as observed after 24 hrs of infection (S9B and S10A Figs).

Taken together, these infection studies with representative mutant strains demonstrate that the intestinal 3D tissue model can recapitulate previously observed phenotypes of known *C*. *jejuni* colonization-determining factors and also reveal phenotypes that are not apparent in 2D-monolayer infections. Although the underlying molecular basis still remains to be identified, opposing phenotypes in 2D vs. 3D were observed for some mutants. Nonetheless, this observation indicates that the tissue model provides the opportunity to compare and/or distinguish requirements of *C*. *jejuni* biology necessary during colonization of human intestinal tissue and various animal models. This in turn can help to unravel pathogen-specific prerequisites that are uniquely necessary in order to colonize the 3D environment of the human intestinal tract.

### Small RNA-mediated regulation of the flagellin modification gene *ptmG* affects *C*. *jejuni* colonization

Based on a comparative RNA-seq study, we had identified more than 40 small regulatory non-coding RNAs (sRNAs) in four *C*. *jejuni* strains [55]. However, their functions and regulatory mechanisms are still largely unknown. Bacterial sRNAs are an emerging class of post-transcriptional gene expression regulators that have also been implicated in virulence control of pathogens [62,63]. Thus, *C*. *jejuni* might likewise depend on its regulatory sRNA repertoire to successfully adapt to different niches in the host ensuring long-term colonization.

One interesting genomic locus encodes a conserved pair of sRNAs, CJnc180/190, which are both abundantly transcribed in multiple *C*. *jejuni* isolates [55]. Recent unpublished work of our group uncovered that the CJnc190 sRNA post-transcriptionally represses expression of Cj1324, a *ptmG* homolog encoded in the *O*-linked flagellin glycosylation island of *C*. *jejuni* strain NCTC11168 (Fig 6C), which is not conserved in 81–176. PtmG is involved in incorporation of legionaminic acid glycan modifications of the flagellin (Fig 6Av) and was shown to play a significant role in the colonization of chickens, but not for interactions with 2D-monolayers [29]. Thus, CJnc190-*ptmG* is an interesting sRNA-target pair for further examination of the utility of the 3D model in exploring *C*. *jejuni* colonization factors and their regulation.

First, we investigated the role of PtmG in ADH and INT in the 3D tissue model vs. 2D-monolayer assays (Fig 6D). Deletion of *ptmG* (Δ*ptmG*) in NCTC11168 had no impact on ADH or INT in a 2D-monolayer, which is in line with a previously reported lack of a phenotype in *in-vitro* human host-cell interactions [29]. However, in the 3D tissue model, the *ptmG*-deficient mutant displayed a two- to three-fold decrease in both ADH (Fig 6D, *upper panel*) as well as INT (Fig 6D, *lower panel*) when compared to the wildtype. This colonization defect could be restored in a complementation strain (C *ptmG*), where *ptmG* is integrated into the unrelated *rdxA* locus of Δ*ptmG* and expressed from its native promoter. The observed colonization defect in 3D seems to be independent of motility, as the Δ*ptmG* strain is fully motile (S8A Fig). Furthermore, approximately two-fold overexpression of *ptmG* by addition of a second gene copy into the parental wild-type strain at the *rdxA* locus (OE *ptmG*) led to increased ADH and INT in the 3D tissue model while having no effect on colonization of non-polarized Caco-2 cells (Fig 6D). This indicates a disparate role for the legionaminic acid flagellin modification on *C*. *jejuni* pathogenesis, depending on infection conditions: the modification is seemingly dispensable for host-pathogen interactions in flat host cells but appears to play an integral role when *C*. *jejuni* comes into contact with the 3D tissue model. This premise is supported by the observation that a *ptmG*-deficient strain is also impeded in its colonization potential of the chicken intestinal tract [29] and shows that the 3D model can reveal phenotypes that are not evident in 2D-monolayers.

Given evidence from our unpublished work that CJnc190 represses *ptmG*, we next tested a ΔCJnc180/190 mutant in our infection model. Compared to wildtype and in contrast to Δ*ptmG*, deletion of CJnc180/190 caused a distinct phenotype in 2D-monolayer infections, where it significantly reduced *C*. *jejuni* ADH (Fig 6D, *upper panel*) and INT (Fig 6D, *lower panel*). These defects were restored to wild-type levels when the sRNA deletion mutant was complemented in *trans* with a wild-type copy of CJnc180/190 expressed from their native promoters (C CJnc180/190), confirming that the observed phenotype is mediated by CJnc180/190. Surprisingly, an opposite phenotype was observed in 3D. *C*. *jejuni* mutants lacking the sRNA pair showed increased ADH to (Fig 6D, *upper panel*) and INT into (Fig 6D, *lower panel*) polarized 3D Caco-2 cells. Again, this phenotype could be restored by complementing sRNA expression in the deletion strain. The seemingly hyper-virulent phenotype in the 3D tissue model of ΔCJnc180/190, in which *ptmG* is upregulated, was reflected by the increased colonization of an overexpression strain of *ptmG*, albeit to a lesser extent. Again, we observed the same trend for these NCTC11168 strains after 4 hrs of infection in 3D as at the 24 hrs time point (S10B Fig). Our observations suggest that at least in part, increased pathogenicity of ΔCJnc180/190 in the 3D tissue model might be due to the regulatory effect of CJnc190 on *ptmG*. However, the results described above also hint towards potential additional targets of CJnc180/190 that might contribute to the observed phenotypes.

Overall, our 3D tissue model proved its ability to uncover phenotypes of colonization and regulatory factors important for host-pathogen interactions of *C*. *jejuni* and reveals a potential role of the CJnc180/190 sRNA pair in virulence of *C*. *jejuni*. Moreover, the 3D tissue model is sensitive enough to detect the importance of factors such as *ptmG*, which seem to be dispensable for host-pathogen interactions with conventionally used non-polarized cells but might play a role in a three-dimensional host environment.

## Discussion

Here, we have established a tissue-engineered, dynamically cultured intestinal 3D infection model, which combines a villi and crypt microarchitecture with an epithelial barrier fortified by cell-cell and cell-ECM interactions, and have employed it to study infections with the enteric pathogen *C*. *jejuni*. Our 3D infection model allows for the investigation of epithelial barrier disruption, bacterial adherence, internalization, as well as transmigration, and is able to identify *in-vivo* relevant infection phenotypes that cannot be observed with non-polarized 2D cell monolayers. Our results demonstrate the power of this infection model for exploring host interactions with *Campylobacter* in a reconstructed 3D tissue environment.

Fluid shear stress has previously been used to stimulate a variety of cell types and proved to be beneficial to their differentiation [64–66]. By applying dynamic culture conditions using an orbital shaker, we could enhance the complexity of the 3D structure of the infection model compared to static culture. This also improved the differentiation of the cells into a columnar-like morphology and the formation of highly organized TJ and AJ complexes. We could show that initial adherence to, internalization into, and transmigration across the 3D tissue model by two *C*. *jejuni* wild-type strains (81–176 and NCTC11168) is decreased/delayed when compared to non-polarized 2D-monolayers and to polarized 2D-Transwells, respectively. This suggests that the ECM scaffold combined with the differentiated 3D structure of the epithelial cells might have an impact on *C*. *jejuni* pathogenicity-determining processes.

The ECM scaffold (SISmuc) retains a high percentage of tightly cross-linked elastin and collagen fibers after decellularization [40,67,68]. *In vivo*, the basal side of enterocytes interacts with the underlying ECM, which in turn could influence the expression and/or localization of cellular receptors and epithelial differentiation [36,69] and thereby the interaction of microbial pathogens with host cells. Moreover, the binding of bacterial surface adhesins to components of the ECM plays a crucial role in colonization of the digestive tract by several other gastrointestinal pathogens such as *Salmonella* Typhimurium and *Helicobacter pylori* [70].

Paracellular transmigration of many gastrointestinal pathogens, such as *S*. Typhimurium, *Shigella flexneri*, *Neisseria gonorrhoeae*, and *Listeria monocytogenes*, is often accompanied by a rapid and severe loss of barrier integrity within the first few hours of infection [52,71,72]. While *C*. *jejuni* reaches the basolateral host cell side in 2D-Transwell-based assays extremely rapidly (within 10–30 min), barrier integrity is not compromised until 8–24 hours of infection [52,73–75]. Similarly, while NCTC11168 and 81–176 were able to cross our dynamically cultured tissue model within 3–4 hours, barrier disruption was not detected until 48 hours p.i. This indicates that *C*. *jejuni* might not induce a permanent opening of AJs and TJs in order to transmigrate. During later time points of infection, we measured an increasing disruption of barrier function and observed a redistribution of cell-cell junction proteins from the lateral membranes to an intracellular location in line with similar observations made with polarized 2D-Transwells and intestinal biopsy samples from campylobacteriosis patients [28,73,74].

Using our 3D tissue model, we could validate the importance of previously observed colonization-determining factors such as the major flagellin FlaA [15,76], or mimic the *in-vivo* relevance of capsular polysaccharide [26,54,77]. For GBS-inducing *C*. *jejuni* strains such as GB11, a potential functional link between ganglioside-like LOS and the presence of a Type II-C CRISPR/Cas system has been suggested [56]. We did not observe any influence on adherence and/or internalization in either 2D or 3D infections for an NCTC11168 *cas9* deletion strain [78]. As NCTC11168 was originally isolated from a gastroenteritis patient without neurological symptoms [79], deletion of *cas9* might not affect colonization of this strain as previously observed for *C*. *jejuni* strain GB11 [56].

*C*. *jejuni* encodes a relatively limited repertoire of regulatory factors for adapting its gene expression to different conditions in the gastrointestinal tract [12,55]. The translational regulator CsrA has been implicated in *C*. *jejuni* colonization of the mouse and the avian intestinal tract, as well as pathogenesis-associated phenotypes such as oxidative stress resistance, biofilm formation, and motility [57,60]. Infection experiments of a Δ*csrA* mutant in *C*. *jejuni* strain 81–176 in our 2D-monolayer and 3D tissue model confirmed the decreased adherence and colonization phenotypes previously reported *in vitro* and *in vivo*, respectively [57,60]. Moreover, we and others [57,60] observed that deletion of *csrA* in this strain results in increased internalization into 2D-monolayers. In contrast, decreased levels of internalized CFUs for this mutant compared to WT were observed in the 3D tissue model. While previous experiments suggest that there is a fairly similar RNA targetome of CsrA in NCTC11168 and 81–176 [58], we cannot exclude the possibility that isolate-specific targets or differences in target expression or affinities for CsrA exist in these diverse strains that could contribute to the different phenotypes observed for their respective *csrA* deletion mutants. Further experiments on the Δ*csrA* transcriptome during infection of the 2D and 3D models might shed light on the signals and expression changes that *C*. *jejuni* requires to adapt to the host environment.

Flagellin glycosylation in a number of Gram-negative bacteria, including *C*. *jejuni* and the related Epsilonproteobacterium *H*. *pylori*, has been reported to play a role in motility, colonization, or interaction with eukaryotic cells [80]. Similar to previous work [29], we did not observe any influence upon deletion of the flagellin modification factor PtmG on *C*. *jejuni* interaction with 2D-monolayers. Remarkably though, in the 3D tissue model, the Δ*ptmG* mutant strain displayed a significant decrease in adherence and internalization. PtmG has previously been shown to be required for successful colonization of chickens [29] and during infection of human volunteers, flagellin glycosylation in general seems to play a role as well [30]. These results highlight the fact that, for example in the case of PtmG, our 3D tissue model was able to reveal an infection phenotype upon its deletion that is not observable with 2D-monolayer experiments, but mimics a colonization defect previously observed *in vivo*. Therefore, the 3D tissue model could be employed as a test system to pre-screen either targeted deletion mutants or genome-wide mutant libraries using *e*.*g*. Tn-seq [81] or to monitor gene expression changes of host and pathogen during the course of infections using Dual RNA-seq [82].

While many non-coding RNAs are differentially regulated during infection, sRNA mutant strains often do not display strong macroscopic infection phenotypes [62,82,83]. Here, we demonstrated that deletion of the *ptmG*-regulating sRNA pair CJnc180/190 increases infection in the 3D tissue model in a way that is consistent with repression of PtmG levels by CJnc190. However, deletion of CJnc180/190 leads to a stronger increase in adherence and internalization compared to an overexpression of *ptmG*. This, together with a decreased infection phenotype of ΔCJnc180/190 in 2D-monolayers that was not mirrored upon overexpression of *ptmG*, suggests that the sRNAs could regulate additional mRNA targets that impact infection. Future studies will reveal these targets of CJnc180/190 and their potential role during *C*. *jejuni* pathogenesis.

In line with our results for *C*. *jejuni* and the tissue-engineered 3D model, various other 3D models including those cultured in the rotating wall vessel bioreactor (RWV) or organs-on-a-chip are promising new tools for infection research and have been applied to study a variety of pathogens, such as *S*. Typhimurium, *Pseudomonas aeruginosa*, or *Mycobacterium tuberculosis* [84–89]. For example, 3D models grown in the RWV successfully recapitulated infection outcomes, such as SPI-1 independent invasion of host cells by *S*. Typhimurium [84], as previously observed *in vivo* [90]. Although our tissue model already successfully recapitulated a 3D host environment and accompanying infection phenotypes, it can still be optimized further. For example, additional complexity could be introduced in the form of a mucus layer, co-culture with other cell types such as immune cells or fibroblasts, or generating them from primary cells. The development of primary organoid cultures derived from stem cells [91] has advanced our understanding of host-pathogen interactions [32]. The spatial orientation of organoids typically requires either dissociation into single cells and seeding in 2D-monolayers [92] or microinjection of the bacteria into the lumen [93] to investigate host-pathogen interactions. Thus, a combination of the organoid/primary cell technology with ECM-based scaffolds offers the chance to develop primary human tissue models, such as recently demonstrated for a 3D intestinal model [40].

Overall, the 3D tissue model described here provides the opportunity to advance our knowledge of molecular factors required during *C*. *jejuni* infection. Moreover, it can readily be applied also for research with other gastrointestinal pathogens. Accordingly, it could provide a bridge between *in-vitro* 2D cell culture systems and animal models, allowing for a more comprehensive analysis of bacterial pathogenesis while simultaneously reducing the need for animal sacrifices along the way.

## Materials and methods

### Cultivation of Caco-2 cells

Human epithelial colorectal adenocarcinoma Caco-2 cells were obtained from the German Collection of Microorganisms and Cell Cultures (DSMZ, Cat. No. ACC 169) and routinely passaged in MEM medium (Gibco) with GlutaMAX and Earle’s salts, supplemented with 20% fetal calf serum (FCS, Biochrom), 1% Non-Essential Amino Acids (NEAA, Gibco), and 1% Sodium Pyruvate (Gibco) in a 5% CO_2_ humidified atmosphere at 37°C. Cells were cultured in T-75 (Sarstedt) flasks until 80–90% confluence and passaged at a sub-culture ratio of 1/2 to 1/10. For passaging, cells were washed once with DPBS (Dulbecco’s phosphate-buffered saline without CaCl_2_ and MgCl_2_, Gibco) to remove residual FCS and subsequently treated with 3 ml of 0.05% Trypsin-EDTA (Gibco) for 5 minutes at 37°C. The trypsinization process was stopped by addition of fresh cell culture medium to the flask and gentle mixing of the cells to give a homogenous single-cell suspension. Depending on the passaging ratio, a certain volume of this suspension was transferred to a new flask containing 10–13 ml of freshly supplemented cell culture medium and cells were given appropriate time to grow to confluence again.

### Ethics statement

For preparation of the decellularized intestinal scaffold (SISmuc), porcine jejunal segments were explanted from six week-old piglets provided by the pig breeder Niedermayer from Dettelbach, Germany. The pigs were sacrificed after heparinization in accordance with the approval given by the government of Lower Franconia in Bavaria, Germany under the study number 55.2-2532-2-256.

Human primary biopsy samples were collected in collaboration with the surgical unit at the University Hospital Würzburg (PD Dr. med. Christian Jurowich, PD Dr. med. Florian Seyfried) from obese adults during routine stomach bypass surgery, and informed consent from patients was obtained beforehand. The use of human primary tissue was approved by the institutional ethics committee on human research of the Julius-Maximilians-University Würzburg under the number 182/10.

### Generation of 3D tissue models and simulation of fluid dynamics

The SISmuc scaffold was generated by decellularization of porcine intestinal tissue via a standardized protocol published previously [35,36]. Briefly, after extensive washing with phosphate-buffered saline (PBS, Gibco), jejunal tissue was subjected to multiple rounds of decellularization with 4% sodium deoxycholate (Sigma Aldrich), alternating with washing steps in PBS. Subsequently, the tissue was digested with a DNase I solution (Roche) and sterilized using gamma radiation (BBFS Sterilisationsservice GmbH, Rommelhausen, Germany). SISmuc scaffolds were kept in this state in PBS at 4°C until usage. In order to generate 3D tissue models, the scaffolds were opened longitudinally, cut into 2 x 2 cm squares, and fixed between two metal rings (so-called cell crowns, Fraunhofer IGB, Stuttgart, Germany). This effectively created an apical and basolateral compartment with the former mucosal surface facing towards the apical side. The surface area of these scaffolds was previously estimated to be 3.1 cm^2^ [36] and was seeded with 3 x 10^5^ Caco-2 cells in 500 μl of the cell culture medium (MEM + 20% FCS + 1% NEAA + 1% Sodium Pyruvate), while the basolateral compartment was filled with 1.5 ml of the same medium. Tissue models were then routinely cultured either statically or dynamically on an orbital shaker (Celltron Infors HT) at 65 rpm in a 5% CO_2_ humidified atmosphere at 37°C for 21–28 days. Fresh medium was supplied every two days. 2D-Transwell inserts (polycarbonate, Ø 12 mm, 0.4/3.0 μm pore size, Corning), were similarly seeded with 3 x 10^5^ Caco-2 cells and cultured statically with medium renewal every second day. Fluid dynamics to determine the rotary frequency of the shaker for dynamic culture conditions were simulated using COMSOL Multiphysics software (Comsol Multiphysics GmbH, Berlin, Germany) and were performed by Ivo Schwedhelm (Tissue Engineering and Regenerative Medicine, University Hospital Würzburg, Germany) as previously described [40].

### Assessment of epithelial barrier integrity

Barrier integrity of the reconstructed mucosal tissue in cell crowns, as well as of confluent cell monolayers on 2D-Transwell inserts during either culture or infection with *C*. *jejuni* was assessed by determining the paracellular flux of fluorescein isothiocyanate-dextran (FITC-dextran). Specifically, 0.25 mg/ml of FITC-dextran (4 kDa, Sigma Aldrich) was resuspended in 500 μl of cell culture medium containing 20% FCS, 1% NEAA, and 1% Sodium Pyruvate, and applied to the apical compartment of tissue models or 2D-Transwells. Prior to that, 1.5 ml of the same culture medium without FITC-dextran was used to fill the basolateral compartment. From both the initial FITC-dextran solution as well as the fresh basolateral cell culture medium, three 100 μl samples were taken as a positive and negative control, respectively, and pipetted into separate wells of a black 96-well plate. Cell culture models were incubated with the FITC-dextran solution in their apical compartments at 37°C for 30 minutes. Afterwards, three 100 μl samples were taken from each basolateral compartment and measured for fluorescence intensity using an Infinite 200 PRO plate reader (TECAN). Autofluorescence of the pure cell culture medium was subtracted from total fluorescence intensities, and FITC-dextran permeability values for each cell culture model were calculated relative to the fluorescence intensity of the initial FITC-dextran solution. Barrier integrity was assessed routinely every seven days during culture. During infection experiments, permeability was measured every 24 hours for the same infected crown as well as for non-infected control cell culture models.

### Histological and immunofluorescence staining and imaging

For histological staining, human small intestinal biopsy samples and 3D tissue models were fixed with 2% PFA (paraformaldehyde, Roth) for at least 1 hour at room temperature (RT), processed for paraffin embedding using a Leica ASP200S tissue processor (Leica Biosystems), and sectioned with 5 μm thickness using a Leica RM2255 microtome (Leica Biosystems). After deparaffinization and rehydration, sections were stained with Hematoxylin and Eosin (H&E; adapted from [94]). After the staining process, light microscopy images were obtained with a Leica DM4000 B microscope (Leica Microsystems).

For immunofluorescence staining, tissue was processed as described above until the deparaffinization and rehydration of the tissue sections was completed. Subsequently, antigens were retrieved by incubation in 10 mM sodium citrate, pH 6 at 90°C for 20 minutes. When staining for *C*. *jejuni*, an additional enzymatic antigen retrieval step was applied by incubation with Proteinase K (Dako) for 12 minutes at RT. Sections were then permeabilized in DPBS with 0.5% Triton X-100 (Roth) for 30 minutes at RT and subsequently blocked with 1% bovine serum albumin (BSA, Roth) in PBS for 30 minutes at RT. Next, sections were incubated overnight at 4°C in a humidity chamber with primary antibody solution (DCS labline), followed by washing 3 x 15 minutes with PBS + 0.05% Tween^20^ (Roth). Subsequently, tissue sections were incubated with the appropriate secondary antibody solution containing DAPI (4′,6-diamidino-2-phenylindole, Sigma Aldrich) for 4 hours at RT in the dark. After three washing steps with PBS + 0.05% Tween^20^ for 15 minutes each at RT, samples were mounted in Mowiol (Sigma Aldrich) and imaged with a laser scanning Leica TCS SP5 II confocal microscope (Leica Microsystems) using z-stack scanning mode. All primary antibodies were diluted 1:100 and included monoclonal mouse anti-E-cadherin (BD Biosciences, 610181), monoclonal mouse anti-occludin (ThermoFisher Scientific, OC3F10), polyclonal rabbit anti-*Campylobacter jejuni* (GeneTex, GTX40882), and polyclonal rabbit anti-cytokeratin-18 (abcam, 1924–1). All secondary antibodies were diluted 1:400 and included goat anti-mouse IgG AlexaFluor555-conjugate (ThermoFisher Scientific, A21424), goat anti-mouse IgG AlexaFluor647-conjugate (ThermoFisher Scientific, A21236), donkey anti-rabbit IgG AlexaFluor594-conjugate (ThermoFisher Scientific, A21207), goat anti-rabbit IgG AlexaFluor647-conjugate (ThermoFisher Scientific, A21246).

### Determination of cell height

For Caco-2 cells grown on 2D-Transwell inserts and SISmuc (static and dynamic culture), as well as for native human intestinal tissues, the mean height of cells was determined as follows. Tissues were fixed with 2% PFA and processed according to the immunofluorescence staining procedure described above. Ten confocal microscopy images were taken for five different Caco-2 cell-based 2D-Transwell inserts and tissue models (static and dynamic culture), respectively. In addition, ten images of similarly processed small intestinal patient samples (n = 5) were used to measure the cell height of every second cell in native tissue. Measurements were made using the program ImageJ. Altogether, 269 cells were measured for Caco-2 cells cultured on 2D-Transwell inserts, 271 cells for statically cultured Caco-2 models, 277 cells for dynamic tissue models, and 199 cells for native human intestine.

### Determination of cell numbers on the Caco-2 3D tissue models

In order to assess the final number of epithelial cells present on a fully developed tissue model (21 days of culture), two methods were employed. First, tissue models were subjected to 30 minutes treatment with 0.05% Trypsin-EDTA at 37°C, cells were detached by scratching with a pipette tip, and then finally counted using a Neubauer counting chamber. The cell numbers for 24 statically cultured and 12 dynamically cultured tissue models were counted (S1 and S3 Tables), respectively. Second, the amount of DNA present in a tissue model was measured using the Quant-iT PicoGreen dsDNA assay kit (ThermoFisher Scientific) according to the manufacturer’s instructions. Briefly, DNA was isolated from 300,000 and 600,000 Caco-2 cells and quantified relative to a previously determined standard curve in order to correlate the DNA amount to a defined number of Caco-2 cells. DNA was also isolated from three statically grown Caco-2 tissue models and three unseeded SISmuc scaffolds and equally quantified. Based on these nucleic acid amounts, cell numbers in the tissue models (minus the residual DNA content of empty scaffolds), were calculated (S2 Table).

### Bacterial strains, oligonucleotides, and plasmids

*C*. *jejuni* strains used in this study are listed in S4 Table. DNA oligonucleotides used for cloning of *C*. *jejuni* mutant strains are listed in S5 Table. Plasmids are listed in S6 Table.

### *C*. *jejuni* standard growth conditions

All *C*. *jejuni* strains were routinely grown at 37°C on Mueller-Hinton (MH, Becton Dickinson) agar plates supplemented with 10 μg/ml vancomycin for 1–2 passages in a HERAcell 150i incubator (ThermoFisher Scientific) in a microaerobic environment (10% CO_2_, 5% O_2_, 85% N_2_). Agar plates were further supplemented with marker-selective antibiotics (20 μg/ml chloramphenicol, 250 μg/ml hygromycin, 50 μg/ml kanamycin) for selection of transformed clones. Bacteria were then transferred to Brucella Broth (BB, Becton Dickinson) liquid cultures in T25 flasks (Corning) by inoculation from plate to a final OD_600_ of 0.005 and grown under agitation at 140 rpm and 37°C.

### Motility assay

Liquid cultures of *C*. *jejuni* strains in BB media containing 10 μg/ml vancomycin were grown under agitation to mid-log phase (OD_600_ 0.4) at 37°C in a microaerobic environment. For each strain, 0.5 μl of bacterial culture was inoculated into a motility soft-agar plate (BB broth + 0.4% Difco agar) poured one day prior to the experiment. Plates were incubated right-side-up until halo formation could be observed (approximately 12–20 hrs post inoculation). For each inoculation, halo radius was measured three times and averaged to give the mean swimming distance for each strain on each plate. Each strain was inoculated in technical triplicates per experiment and motility assays were performed in three independent biological replicates. The average halo radius for each strain was used to compare motility between *C*. *jejuni* wild-type and mutant strains.

### Construction of *C*. *jejuni* deletion mutant strains

Deletion mutants of *C*. *jejuni* used in this study were constructed by double-crossover homologous recombination with antibiotic resistance cassettes to remove most of the coding sequence in the genomic locus thereby disrupting the respective genes. Resistance cassettes used for cloning were either *aphA-3* (Kan^R^) [95], *aph(7”)* (Hyg^R^) [96], or *cat*.*coli* (Cm^R^) [97]. Non-polar resistance cassettes were amplified with primers HPK1/HPK2 from plasmid pGG1 [58] for Kan^R^, with primers CSO-1678/CSO-1679 from plasmid pAC1H [96] for Hyg^R^, or primers CSO-0613/0614 from *C*. *jejuni* strain CSS-0643 [58] for Cm^R^. Overlap PCR products carried these resistance cassettes flanked by ~500 bp of homologous sequence up- and downstream of the gene to be deleted. As an example, deletion of *kpsMT* in *C*. *jejuni* strain NCTC11168 (CSS-0032) will be described in detail. First, ~500 bp upstream of the *kpsM* (Cj1448c) start codon using CSO-2009 and CSO-2008 and ~500 bp downstream of the *kpsM* stop codon using CSO-2011 and CSO-2010 were amplified from genomic DNA of NCTC11168 wild-type strain. As the start codon of the downstream gene *kpsT* (Cj1447c) overlaps with the stop codon of *kpsM*, the mutant created here likely resulted in the inactivation of *kpsT* as well. The kanamycin resistance cassette (*aphA-3*) was amplified from pGG1 using HPK1 and HPK2. To fuse the up- and downstream region of *kpsM* with the *aphA-3* resistance cassette, the antisense oligonucleotide of the *kpsM* upstream region (CSO-2008) contained 22 bp of overlap with the sense oligonucleotide used to amplify the *aphA-3* resistance cassette (HPK1). Likewise, the sense oligonucleotide of the *kpsM* downstream region (CSO-2011) contained 26 bp overlap with the antisense oligonucleotide to amplify the *aphA-3* resistance cassette (HPK2). In a final 100 μl Phusion polymerase PCR reaction, the purified (Macherey-Nagel NucleoSpin PCR cleanup kit) up- and downstream regions of *kpsM* were added together with the *aphA-3* resistance cassette in a ratio of 50:50:90 ng and amplified using CSO-2009 and CSO-2011 (final concentration of 1 μM). The program for the overlap PCR was as follows: 1 cycle of [98°C, 1 min; 61°C, 1 min; 72°C, 10 min; 98°C, 1 min], 40 cycles of [98°C, 15 s; 57°C, 30 s; 72°C, 1 min], followed by 72°C for 10 min. Overlap PCR products were verified for their size by agarose gel electrophoresis and after purification subsequently transformed into the recipient *C*. *jejuni* strain by electroporation (see protocol described below). After verification of the resulting clones via colony PCR with CSO-2008 and HPK2, a positive clone was picked for the final *kpsMT* deletion strain (CSS-6198; NCTC11168 Δ*kpsMT*). Deletion mutants for *flaA* (CSS-1512) [58], *csrA* (CSS-0643) [58], *cas9* (CSS-3836) [78], and *ptmG* (CSS-2966) in *C*. *jejuni* strain NCTC11168, as well as for *flaA* (CSS-2380), *kpsMT* (CSS-6200), and *csrA* (CSS-6202) in strain 81–176 (CSS-0063) were constructed using an analogous approach and oligonucleotides are listed in S5 Table.

The sRNA locus CJnc180/190 was deleted in the same way as described with the exception of using a resistance cassette that included a promoter and a terminator sequence. This resistance cassette was amplified using JVO-5068 and HPK2term from pGG1 and annealed together with the purified up- and downstream fragments of CJnc180/190. The up- and downstream regions of CJnc180/190 were amplified using CSO-0247/CSO-0248 and CSO-0249/CSO-0250, respectively, from wild-type genomic DNA of strain NCTC11168. CSO-0248 and CSO-0249 contained overlapping regions to the polar kanamycin resistance cassette with a promoter and terminator region from the *H*. *pylori* sRNA RepG [98]. The upstream region and downstream region as well as cassette PCR amplicons were then annealed and the entire product was amplified with CSO-0247 and CSO-0250 and electroporated into the *C*. *jejuni* NCTC11168 wild-type strain. Kanamycin-resistant colonies were validated via colony PCR using CSO-0246 and HPK1, resulting in CJnc180/190::*aphA-3* (CSS-1157).

### Construction of *C*. *jejuni* complementation and overexpression strains

In order to complement the deletion of a gene or create an overexpression construct, a wild-type copy of the gene of interest was inserted into the *rdxA* (Cj1066) locus, which is frequently used for complementation in *C*. *jejuni* [99]. To achieve this, complementation/overexpression constructs were first generated in plasmids that contained approximately 500 bp up- and downstream sequences of the insertion site in the *rdxA* gene, flanking Cm^R^ or Kan^R^ resistance cassettes that each contained a promoter and terminator, or were generated by overlap PCR.

As an example for the plasmid-based approach, the construction of the *ptmG* complementation and overexpression in strain NCTC11168 is described in detail. The coding region of *ptmG* including approximately 100 nt up- and downstream of the start and stop codon, respectively, was amplified from genomic DNA of *C*. *jejuni* strain NCTC11168 using CSO-2928 and CSO-2929 and digested with *Xma*I and *Pst*I. The backbone for the *ptmG* complementation/overexpression vector was amplified by inverse PCR with CSO-0762 and CSO-0493 from pST1.1 [78] and likewise digested (*Xma*I/*Pst*I). The plasmid backbone and the insert were ligated and transformed into *E*. *coli* TOP10. The clones were verified by colony PCR using CSO-0023 and CSO-2929 and sequenced using CSO-0023 (Macrogen), resulting in pSSv63.1. A purified complementation construct, amplified from pSSv63.1 with CSO-2276 and CSO-2277, was then transformed into *C*. *jejuni* strain NCTC11168 Δ*ptmG* (CSS-2966) via electroporation for complementation or into NCTC11168 WT (CSS-0032) for overexpression of *ptmG*. The final complementation/overexpression clones were verified by colony PCR using CSO-0023 and CSO-0349 and sequencing using CSO-0023, resulting in the complementation strain C *ptmG* (CSS-2978; *ptmG*::*aph(7”)*; *rdxA*::*aphA-3-ptmG*) and overexpression strain OE *ptmG* (CSS-2980; *rdxA*::*aphA-3-ptmG*), respectively. An analogous approach was carried out for C CJnc180/190 (CSS-1158; CJnc180/190::*aphA-3*, *rdxA*::*cat*.*coli*-CJnc180/190) in their respective deletion mutant strains.

Generation of complementation constructs by overlap PCR (i.e., *rxdA*::*cat*.*coli-flaA*, *rdxA*::*cat*.*coli-kpsMT*, and *rdxA*::*aphA-3-csrA*) was performed as follows, using NCTC11168 *flaA* as an example. A fragment encoding the *rdxA* upstream region (approximately 500 bp) fused to the *cat*.*coli* Cm^R^ resistance cassette (with promoter and terminator) was amplified by PCR from pGD34.7 using CSO-2276/CSO-0573, and the *rdxA* downstream region was amplified with CSO-0347/CSO-2277 from NCTC11168 wild-type genomic DNA. The *flaA* gene, with its native promoter [55], was amplified from NCTC11168 wild-type genomic DNA using CSO-4744/CSO-4745, such that the 5’-end of the amplicon had complementarity to CSO-0573, and the 3’-end of the amplicon overlapped with CSO-0347. The *rdxA*_up-*cat*.*coli*, *flaA* insert, and *rdxA*_down fragments were then annealed, and the entire product was amplified with CSO-2276 and CSO-2277. The resulting PCR product was electroporated into *C*. *jejuni* NCTC11168 Δ*flaA*, and insertion mutants were validated by colony PCR with CSO-0643/CSO-0349 and sequencing with CSO-0643/CSO-4474/CSO-3270. The same approach was used for complementation of Δ*kpsMT* strains. For complementation of Δ*csrA*, a similar approach was used, except the upstream fragment encoded *rdxA*_up-*aphA-3*. This fragment was amplified from pST1.1 with CSO-2276/CSO-0762. The *csrA* insert for both strains included a putative promoter encoded within the upstream *truB* locus, identified by differential RNA-seq [55]. Complemented *csrA* strains were validated by colony PCR with CSO-0023/CSO-3270 and sequencing with CSO-0023.

### Transformation of *C*. *jejuni* by electroporation

*C*. *jejuni* NCTC11168 or 81–176 wildtype or appropriate deletion mutants were streaked onto MH agar plates with the suitable antibiotics from cryostocks. After one passage, bacterial cells were harvested with a cotton swab and resuspended in cold electroporation solution (272 mM sucrose, 15% (w/v) glycerol). Bacteria were harvested by centrifugation at 4°C and 6,500 x *g* for 5 minutes, and then resuspended in the same solution. After two additional washing steps, the final pellet was resuspended in an appropriate small volume of electroporation solution, depending on the size of the pellet. Next, 50 μl of this cell suspension was mixed with 200–400 ng of purified PCR product (not exceeding 4 μl in total) and electroporated (Biorad Genepulser) in a 1 mm gap cuvette (PEQLAB) at 2.5 kV, 200 Ω, and 25 μF. By adding 200 μl prewarmed Brucella Broth, cells were then transferred onto a non-selective MH agar plate and recovered overnight at 37°C in a microaerobic environment. The next day, bacterial cells were harvested with a cotton swab, streaked onto an appropriate selective MH agar plate, and incubated at 37°C microaerobically until colonies were observed (typically 2–4 days). Clones were verified by colony PCR and sequencing, cryostocks were frozen in 25% glycerol in BB medium, and stored at -80°C.

### Infection of the 3D tissue model with *C*. *jejuni*

Caco-2 cell-based tissue models were cultured either statically or dynamically for 21 days before they were used for infection experiments with *C*. *jejuni*. Independent of pre-culture of the tissue models, infection with *C*. *jejuni* was always conducted under static conditions without additional mechanical stimulation. Bacteria were grown as described above, harvested from liquid culture in mid-log phase (OD_600_ 0.4), and resuspended in fresh cell culture medium containing 20% FCS, 1% NEAA, and 1% Sodium Pyruvate to achieve a multiplicity of infection (MOI) of 20. From this bacterial cell suspension, serial dilutions were plated onto MH agar plates and incubated at 37°C under microaerobic conditions to determine the input amount of colony forming units (CFUs). 500 μl of these bacterial suspensions were used to apically infect the tissue models and co-incubation was carried out in a 5% CO_2_ humidified atmosphere at 37°C. For transmigration experiments, 100 μl samples from the basolateral compartment of infected tissue models were taken at indicated time points post infection (10 min to 8 hrs p.i.) and serial dilutions were plated on MH agar plates to determine the number of transmigrated bacteria. To isolate bacteria adherent to and internalized into the tissue models, infection was stopped at indicated time points post infection (4–120 hrs). For experiments involving longer time points (24–120 hrs), spent cell culture medium was exchanged daily for fresh cell culture medium supplemented with 20% FCS, 1% NEAA, and 1% Sodium Pyruvate in the apical and the basolateral compartment of the tissue models. To harvest cell-associated bacteria, cell crowns were washed three times with DPBS to remove all non-adherent bacterial cells. Subsequently, two tissue pieces per crown were collected using a tissue punch (Ø 5 mm, Kai Medical) and transferred to an Eppendorf tube with 500 μl of 0.1% saponin in DPBS. The tissue pieces were incubated for 10 minutes at 37°C under agitation to isolate bacteria from host cells. Serial dilutions were then plated on MH agar plates, colonies were counted, and CFU numbers were calculated as a percentage of input CFUs for each strain. In order to specifically isolate host cell-internalized *C*. *jejuni*, fresh cell culture medium with 20% FCS, 1% NEAA, and 1% Sodium Pyruvate containing 200 μg/ml gentamicin was supplied to the basolateral and the apical compartment of the tissue models to kill extracellular bacteria for 2 hours at 37°C. This concentration of gentamicin was determined by subjecting both *C*. *jejuni* 81–176 and NCTC11168 to varying concentrations of gentamicin (10–400 μg/ml) for different periods of time (30–240 min). Plating for CFUs was used as a readout for bacteria surviving the antibiotic treatment. After treatment with 200 μg/ml for 2 hrs, no colonies could be recovered for either of the wild-type strains. In addition, CFU plating of the supernatant for each *C*. *jejuni* strain after this gentamicin treatment ensured that no extracellular bacteria had survived. For experiments involving intracellular survival of *C*. *jejuni* in the tissue model, the initial treatment with 200 μg/ml of gentamicin for 2 hrs was followed by replacement of the medium in the basolateral and the apical compartment for fresh cell culture medium containing 20% FCS, 1% NEAA, and 1% Sodium Pyruvate supplemented with 10 μg/ml gentamicin. Again, CFU plating of the supernatant ensured that no extracellular bacteria could be recovered after this treatment. After gentamicin treatment, tissue models were washed three times with DPBS before CFU numbers were determined as described above.

### Infection of 2D cell culture models (2D-monolayer and 2D-Transwell) with *C*. *jejuni*

In general, infection experiments were carried out as described for 3D tissue models with a few modifications. For 2D-monolayer infections, Caco-2 cells were seeded into 6-well plates two days prior to the infection experiment in order to achieve a confluent cell monolayer. *C*. *jejuni* was grown in liquid culture to mid-log phase, resuspended in cell culture medium and used for infection of the epithelial monolayer at an MOI of 20. After infection, cells were washed three times with DPBS, lysed with 0.1% saponin in 1 ml DPBS, and the resulting cell suspension was plated in serial dilutions on MH agar plates. For specific recovery of intracellular bacteria, cells were treated with 200 μg/ml gentamicin for 2 hrs at 37°C and CFUs were determined as described. In addition, CFU plating of the supernatant of gentamicin-treated wells ensured that all extracellular bacteria were killed during the antibiotic treatment. For 2D-Transwell infections, Caco-2 cells were grown on polycarbonate 2D-Transwell inserts (Corning, 12 mm, 3.0 μm) for 21 days in a 5% CO_2_ humidified atmosphere at 37°C. Isolation and enumeration of CFU numbers of colonizing or transmigrated bacteria was carried out the same way as described above for the 3D tissue models.

### Growth curve analysis in cell culture medium and infection supernatants

To analyze the growth behavior of *C*. *jejuni* WT strains in tissue culture medium, bacteria were first routinely grown on MH agar plates and subsequently in BB liquid cultures overnight as described above. The next morning, bacterial cells were washed once with PBS and inoculated to a final OD_600_ of 0.05 into 50 ml cell culture medium (MEM + 20% FCS, 1% NEAA, 1% Sodium Pyruvate) and grown under agitation at 140 rpm in a HERAcell 150i incubator (ThermoFisher Scientific) in a microaerobic environment (10% CO_2_, 5% O_2_, 85% N_2_). Samples were removed at appropriate time-points post-inoculation for plating on MH agar to determine CFUs/ml. To measure growth of *C*. *jejuni* wild-type strains in the supernatant above 3D tissue models and 2D-Transwells, infection experiments with *C*. *jejuni* strains NCTC11168 and 81–176 were carried out as described above with the exception that the cell culture medium was not exchanged daily. At the appropriate time points post-inoculation, 10 μl samples were taken from the supernatant for plating on MH agar plates to ascertain CFUs/ml.

## Supporting information

S1 Fig Static and perfusion bioreactor culture of Caco-2 cells on the SISmuc scaffold. **(A)** Images of the static (*upper panel*) and perfusion bioreactor (*lower panel*) conditions for the establishment of 3D tissue models. Under static conditions, metal cell crowns containing the reconstructed tissue are cultured in conventional 12-well plates without additional mechanical stimulation. The perfusion bioreactor enables the culture of tissue models under a continuous medium flow over the cell surface leading to stimulating shear stress on the cells. **(B)** Hematoxylin and Eosin (H&E) (*upper panel*) staining of human small intestinal tissue (*left panel*) or Caco-2 cells on SISmuc after 21 days in static (*middle panel*) or perfusion bioreactor culture (*right panel*). The same samples were stained with DAPI (nuclei, blue) and an antibody against ZO-1 (zonula occludens-1, green) to detect the development of tight junctions during the respective culture conditions. The tissue was fixed with 2% PFA, processed for paraffin embedding and sectioned with 5 μm thickness. Scale bars for H&E: 200 μm; scale bars for IHC: 25 μm.
(TIF)

S2 Fig Dynamic culture of Caco-2 cells grown on SISmuc scaffold promotes cell height. Determination of the average cell height for Caco-2 cells cultured on 2D-Transwell inserts or SISmuc (static and dynamic conditions) compared to native human intestine. Ten confocal microscopy images each of five different 2D-Transwells or 3D tissue models based on Caco-2 cells (static and dynamic conditions) and five different patient samples were used to measure the cell height of every second cell in the pictures using ImageJ. Asterisks above each bar indicate the statistical significance between human intestine and Caco-2 cells on 2D-Transwells, Caco-2 cells on SISmuc (static), or Caco-2 cells on SISmuc (dynamic). ****: *p* < 0.0001, using Student’s *t*-test.
(TIF)

S3 Fig Set-up of infection readouts and isolation of bacterial CFUs. **(A I-III)** After infection of the 3D tissue model with *C*. *jejuni* (MOI 20), cell crowns are investigated for **(I)** bacterial burden by isolation of CFUs using a tissue punch (5 mm diameter) and enumeration of CFUs by serial dilution on agar plates, for **(II)** disruption of epithelial barrier function by FDPA, or for **(III)** phenotypic characteristics by confocal microscopy analyses after immunohistochemical staining (IHC). **(B)** Detergent test for isolation of CFUs from the tissue models. Recovery of CFUs (adherent + internalized) from the static Caco-2 cell-based tissue model 24 hrs p.i. with *C*. *jejuni* wild-type strains NCTC11168 and 81–176. Different concentrations (0.01%, 0.1%, and 1.0%) of saponin (*left panel*) and Triton X-100 (*right panel*) were tested for their efficiency to isolate bacteria from infected tissue. **: *p* < 0.01, *: *p* < 0.05, ns: not significant, using Student’s *t*-test.
(TIF)

S4 Fig Colonization and disruption of epithelial barrier function by *C*. *jejuni* in the 2D-Transwell system. **(A)** Colonization of 2D-Transwells by *C*. *jejuni* strains NCTC11168 and 81–176 from 24–120 hrs p.i. CFUs are represented as the mean value of three independent experiments with corresponding SDs and are depicted as the percentage of input CFUs. Statistical difference was calculated for the comparison between the two wild-type strains. **(B)** FDPA measurements were conducted to determine disruption of epithelial barrier function of 2D-Transwells, which were either left untreated (mock) or infected for up to 120 hrs with *C*. *jejuni* strain NCTC11168 or 81–176. FDPA values represent the mean of three biological replicates with corresponding SDs and are depicted as fold changes relative to time point zero. Based on these fold changes, statistical significance was calculated between the two wild-type strains for each time point, as well as between NCTC11168/81-176 and the non-infected control at 24 hrs p.i. ****: *p* < 0.0001, ***: *p* < 0.001, **: *p* < 0.01, *: *p* < 0.05, ns: not significant, using Student’s *t*-test.
(TIF)

S5 Fig Growth of *C*. *jejuni* wild-type strains in cell culture medium and infection model supernatant. **(A, B)** Replication of *C*. *jejuni* wild-type strains in cell culture medium (MEM + 20% FCS, 1% NEAA, 1% Sodium Pyruvate) supernatant of 2D-Transwells (*upper panel*) and 3D tissue models (*lower panel*) during the course of infection **(A)** or in cell culture medium alone **(B)**. CFUs are depicted as CFUs/ml (x10^7^) and represent the mean of three independent experiments with respective SDs. Statistical significance in **(A)** and **(B)** is calculated for the comparison between wild-type strains. **: *p* < 0.01, ns: not significant, using Student’s *t*-test.
(TIF)

S6 Fig *C*. *jejuni* infection of the 3D tissue models leads to mislocalization of occludin. Confocal microscopy images of paraffin sections of the Caco-2 cell-based 3D tissue model cultured dynamically during infection with *C*. *jejuni* strains 81–176 and NCTC11168 (24–120 hrs p.i.) or non-infected controls. Bacteria were detected with an anti-*C*. *jejuni* antibody (green), nuclei were stained with DAPI (blue), and an anti-occludin antibody was used to visualize TJs (tight junctions, magenta). White arrows indicate regions of redistribution of tight junction staining from the periphery of the cell to intracellular regions as well as loss of apical staining for occludin. Images in the second row for each strain are 3-fold magnifications of the indicated region in the respective confocal image above. Scale bars: 10 μm.
(TIF)

S7 Fig Internalization and intracellular survival of *C*. *jejuni* in the 3D tissue model. **(A)** Internalization of *C*. *jejuni* NCTC11168 and 81–176 WT strains into the 3D tissue model was determined at each time point after a 2 hrs gentamicin treatment (200 μg/ml) with subsequent isolation of CFUs. Experiments were performed in triplicates and internalized CFUs (percentage of input) are depicted as the mean with corresponding SDs. **(B)** To determine intracellularly surviving bacteria, 3D tissue models infected with *C*. *jejuni* NCTC11168 and 81–176 were treated with 200 μg/ml gentamicin for 2 hrs at the 24 hrs time point only. Subsequently, medium in both apical and basolateral compartments was exchanged for fresh cell culture medium containing 10 μg/ml gentamicin to inhibit growth of bacteria released from host cells. CFUs were recovered at the indicated time points to determine the number of surviving intracellular bacteria and are depicted as the mean of three biological replicates with respective SDs (percentage of input). Statistical significance in both **(A)** and **(B)** was calculated for the comparison of CFUs between strains NCTC11168 and 81–176. ***: *p* < 0.001, **: *p* < 0.01, ns: not significant, using Student’s *t*-test.
(TIF)

S8 Fig Motility assay of *C*. *jejuni* WT and mutant strains. **(A)** *C*. *jejuni* NCTC11168 wildtype, deletion mutants (Δ*flaA*, Δ*kpsMT*, Δ*cas9*, Δ*csrA*, Δ*ptmG*, ΔCJnc180/190), respective complementation strains (C *flaA*, C *kpsMT*, C *cas9*, C *ptmG*, C CJnc180/190), and the overexpression mutant OE *ptmG* were grown overnight in Brucella broth (BB) liquid culture to mid-log phase (OD_600_ 0.4) and subsequently stabbed into 0.4% soft agar BB plates. After 24 hrs of incubation at 37°C in a microaerobic environment, motility was measured by determining the swimming halo radius in comparison to wild-type behavior. **(B)** *C*. *jejuni* 81–176 wildtype, deletion mutants (Δ*flaA*, Δ*kpsMT*, Δ*csrA*), and respective complementation mutants (C *flaA*, C *kpsMT*, C *csrA*) were assessed for motility as described for NCTC11168 strains above. Bar graphs and corresponding SDs represent the mean of three biological replicates. Statistical significance was calculated for the comparison between each mutant strain and its respective wildtype. ****: *p* < 0.0001, *: *p* < 0.05, ns: not significant, using Student’s *t*-test.
(TIF)

S9 Fig Infection outcomes of *C*. *jejuni* 81–176 deletion mutants in 2D-monolayer and 3D tissue model infections. **(A, B)** Adherence (*upper panels*) and internalization (*lower panels*) of *C*. *jejuni* 81–176 wildtype (WT), deletion mutants (Δ*flaA*, Δ*kpsMT*, Δ*csrA*), and their respective complementation strains (C *flaA*, C *kpsMT*, C *csrA*) was examined at 4 hrs p.i. in 2D-monolayers and 24 hrs p.i. in 3D tissue models **(A)** as well as 4 hrs p.i. in 3D tissue models **(B)**. CFUs are depicted as a percentage of input and represent the mean of three biological replicates with corresponding SDs. Asterisks or ns above each bar indicate the significance of the tested mutant strain compared to their respective wildtype in 2D or 3D. ****: *p* < 0.0001, ***: *p* < 0.001, **: *p* < 0.01, *: *p* < 0.05, ns: not significant, using Student’s *t*-test.
(TIF)

S10 Fig Infection phenotypes of NCTC11168 mutants in the 3D tissue model at 4 hrs p.i. **(A, B)** Isolation of CFUs from 3D tissue models (4 hrs p.i.) infected with *C*. *jejuni* NCTC11168 wildtype (WT) and deletion/complementation mutants from Main Fig 6 for either adherence (*upper panels*) or internalization (*lower panels*). CFUs are depicted as the percentage of their respective input CFUs and represent the mean of three biological replicates with corresponding SDs. Asterisks or ns above each bar indicate the significance of the tested mutant strain compared to their respective wildtype in 3D. ****: p < 0.0001, ***: p < 0.001, **: p < 0.01, *: p < 0.05, ns: not significant, using Student’s *t*-test.
(TIF)

S1 Table Cell counting of statically cultured tissue models. Caco-2 cells of statically cultured 3D tissue models were harvested by trypsin treatment coupled with extensive mechanical dissolution. Subsequently, cells were counted in a Neubauer counting chamber using the trypan blue exclusion method.
(DOCX)

S2 Table PicoGreen assay of statically cultured tissue models. DNA content of 300,000 and 600,000 Caco-2 cells as well as three statically cultured 3D tissue models was determined using the Quant-iT PicoGreen dsDNA assay kit. The relative fluorescence intensities designated with an asterisk indicate the final intensity after subtraction of background fluorescence of unseeded SISmuc.
(DOCX)

S3 Table Cell counting of dynamically cultured tissue models. As for the static tissue models (S1 Table), Caco-2 cells of dynamically cultured 3D tissue models were harvested and counted.
(DOCX)

S4 Table Bacterial strains. (DOCX)

S5 Table DNA oligonucleotides. (DOCX)

S6 Table Plasmids. (DOCX)
