# Supplementary figures and images for: A three-dimensional intestinal tissue model reveals factors and small regulatory RNAs important for colonization with Campylobacter jejuni
Source: PLoS Pathog. 2020 Feb 18;16(2):e1008304. doi: 10.1371/journal.ppat.1008304 (PMC7048300; doi:10.1371/journal.ppat.1008304)

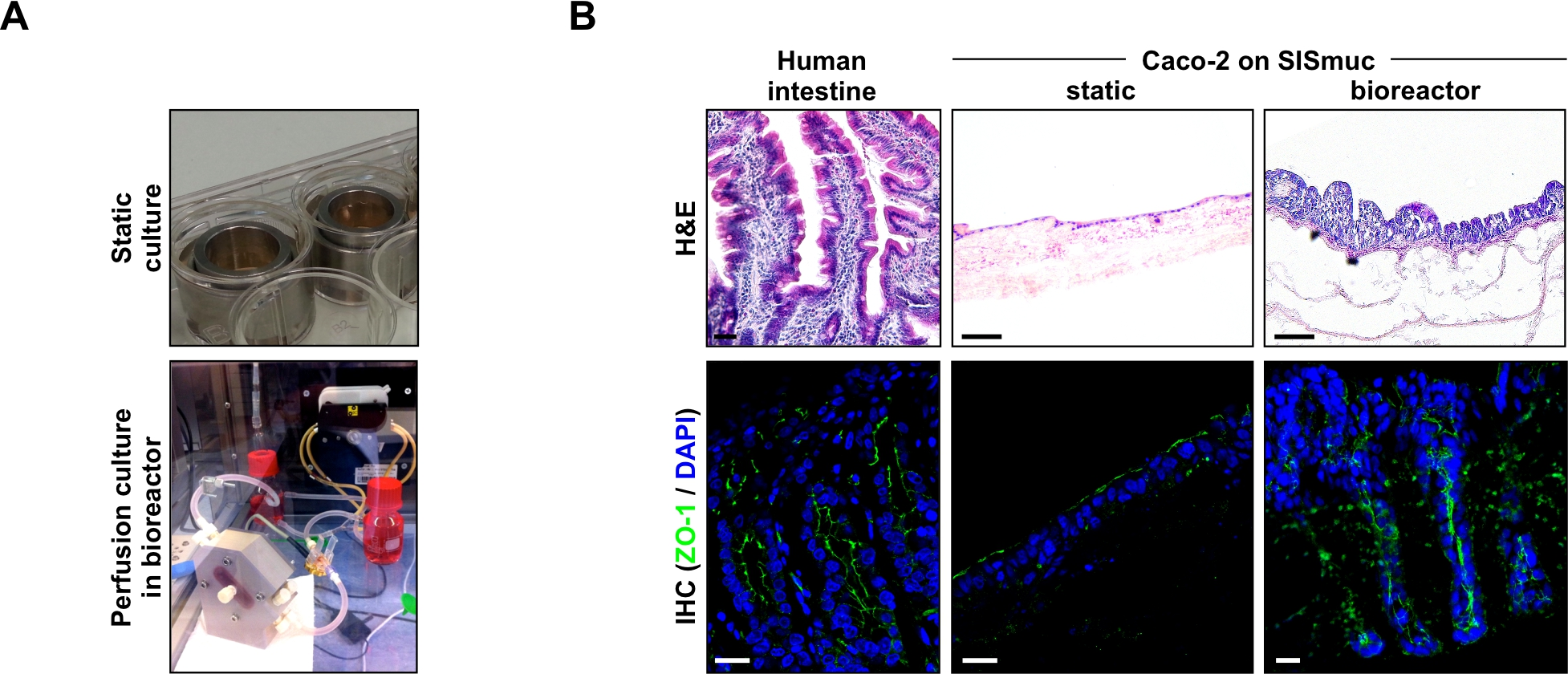

Supplement: S1 Fig — (A) Images of the static (upper panel) and perfusion bioreactor (lower panel) conditions for the establishment of 3D tissue models. Under static conditions, metal cell crowns containing the reconstructed tissue are cultured in conventional 12-well plates without additional mechanical stimulation. The perfusion bioreactor enables the culture of tissue models under a continuous medium flow over the cell surface leading to stimulating shear stress on the cells. (B) Hematoxylin and Eosin (H&E) (upper panel) staining of human small intestinal tissue (left panel) or Caco-2 cells on SISmuc after 21 days in static (middle panel) or perfusion bioreactor culture (right panel). The same samples were stained with DAPI (nuclei, blue) and an antibody against ZO-1 (zonula occludens-1, green) to detect the development of tight junctions during the respective culture conditions. The tissue was fixed with 2% PFA, processed for paraffin embedding and sectioned with 5 μm thickness. Scale bars for H&E: 200 μm; scale bars for IHC: 25 μm. (TIF) [file ppat.1008304.s001.tif]

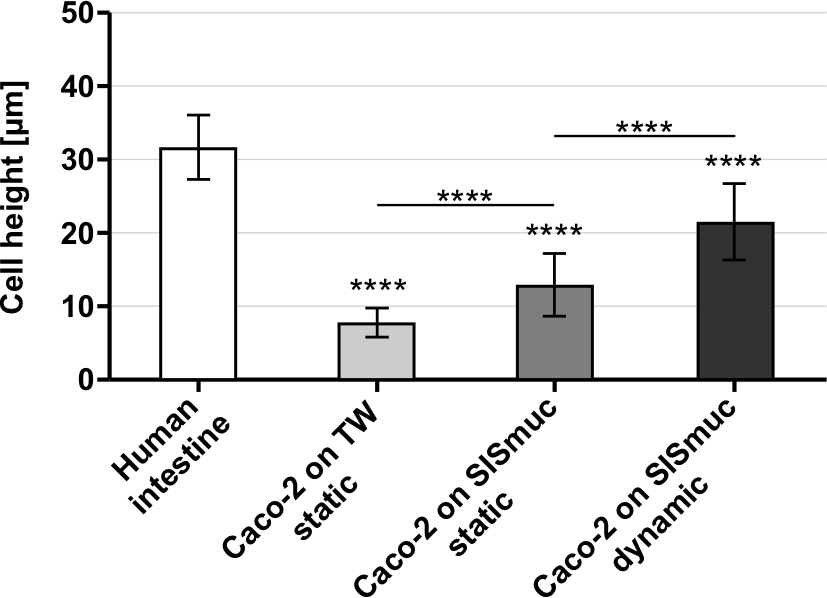

Supplement: S2 Fig — Determination of the average cell height for Caco-2 cells cultured on 2D-Transwell inserts or SISmuc (static and dynamic conditions) compared to native human intestine. Ten confocal microscopy images each of five different 2D-Transwells or 3D tissue models based on Caco-2 cells (static and dynamic conditions) and five different patient samples were used to measure the cell height of every second cell in the pictures using ImageJ. Asterisks above each bar indicate the statistical significance between human intestine and Caco-2 cells on 2D-Transwells, Caco-2 cells on SISmuc (static), or Caco-2 cells on SISmuc (dynamic). ****: p < 0.0001, using Student’s t-test. (TIF) [file ppat.1008304.s002.tif]

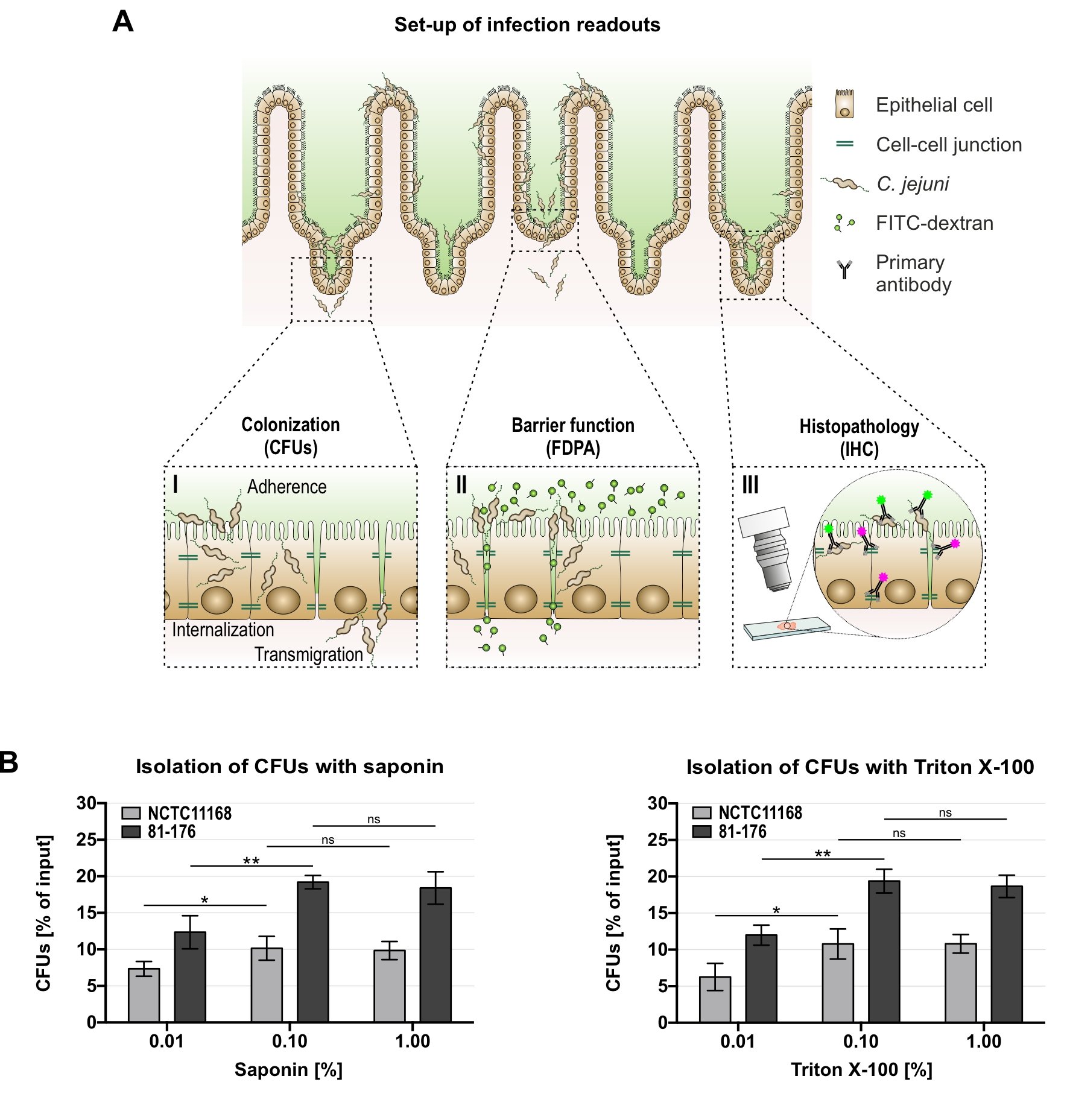

Supplement: S3 Fig — (A I-III) After infection of the 3D tissue model with C. jejuni (MOI 20), cell crowns are investigated for (I) bacterial burden by isolation of CFUs using a tissue punch (5 mm diameter) and enumeration of CFUs by serial dilution on agar plates, for (II) disruption of epithelial barrier function by FDPA, or for (III) phenotypic characteristics by confocal microscopy analyses after immunohistochemical staining (IHC). (B) Detergent test for isolation of CFUs from the tissue models. Recovery of CFUs (adherent + internalized) from the static Caco-2 cell-based tissue model 24 hrs p.i. with C. jejuni wild-type strains NCTC11168 and 81–176. Different concentrations (0.01%, 0.1%, and 1.0%) of saponin (left panel) and Triton X-100 (right panel) were tested for their efficiency to isolate bacteria from infected tissue. **: p < 0.01, *: p < 0.05, ns: not significant, using Student’s t-test. (TIF) [file ppat.1008304.s003.tif]

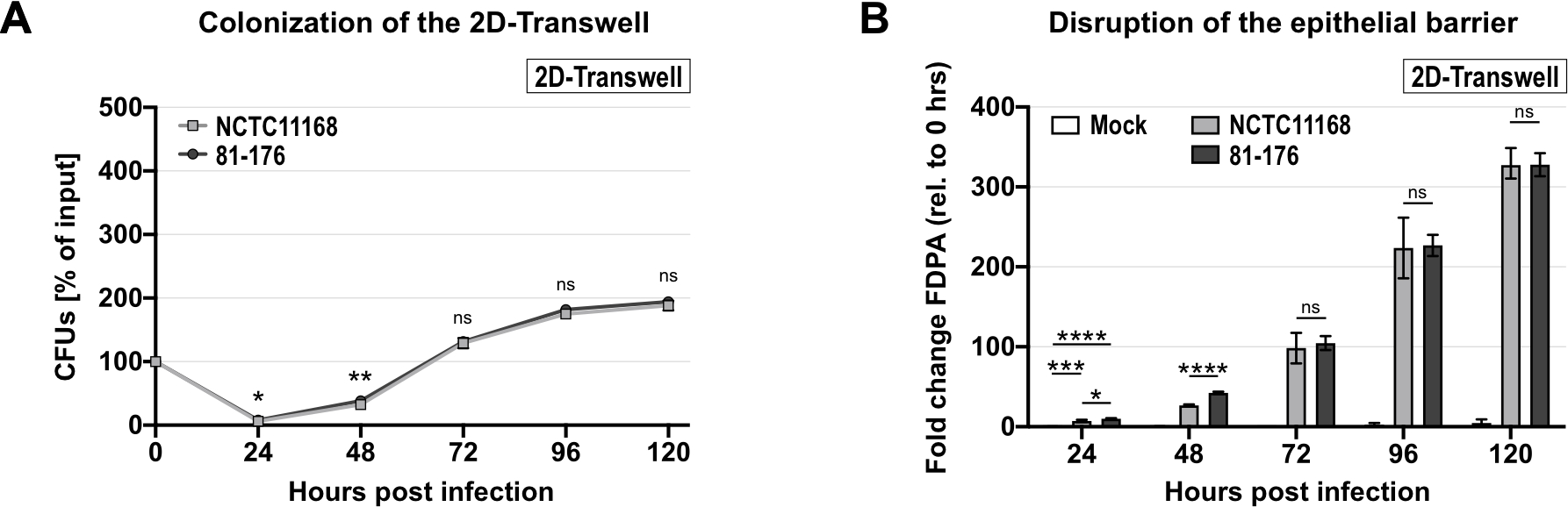

Supplement: S4 Fig — (A) Colonization of 2D-Transwells by C. jejuni strains NCTC11168 and 81–176 from 24–120 hrs p.i. CFUs are represented as the mean value of three independent experiments with corresponding SDs and are depicted as the percentage of input CFUs. Statistical difference was calculated for the comparison between the two wild-type strains. (B) FDPA measurements were conducted to determine disruption of epithelial barrier function of 2D-Transwells, which were either left untreated (mock) or infected for up to 120 hrs with C. jejuni strain NCTC11168 or 81–176. FDPA values represent the mean of three biological replicates with corresponding SDs and are depicted as fold changes relative to time point zero. Based on these fold changes, statistical significance was calculated between the two wild-type strains for each time point, as well as between NCTC11168/81-176 and the non-infected control at 24 hrs p.i. ****: p < 0.0001, ***: p < 0.001, **: p < 0.01, *: p < 0.05, ns: not significant, using Student’s t-test. (TIF) [file ppat.1008304.s004.tif]

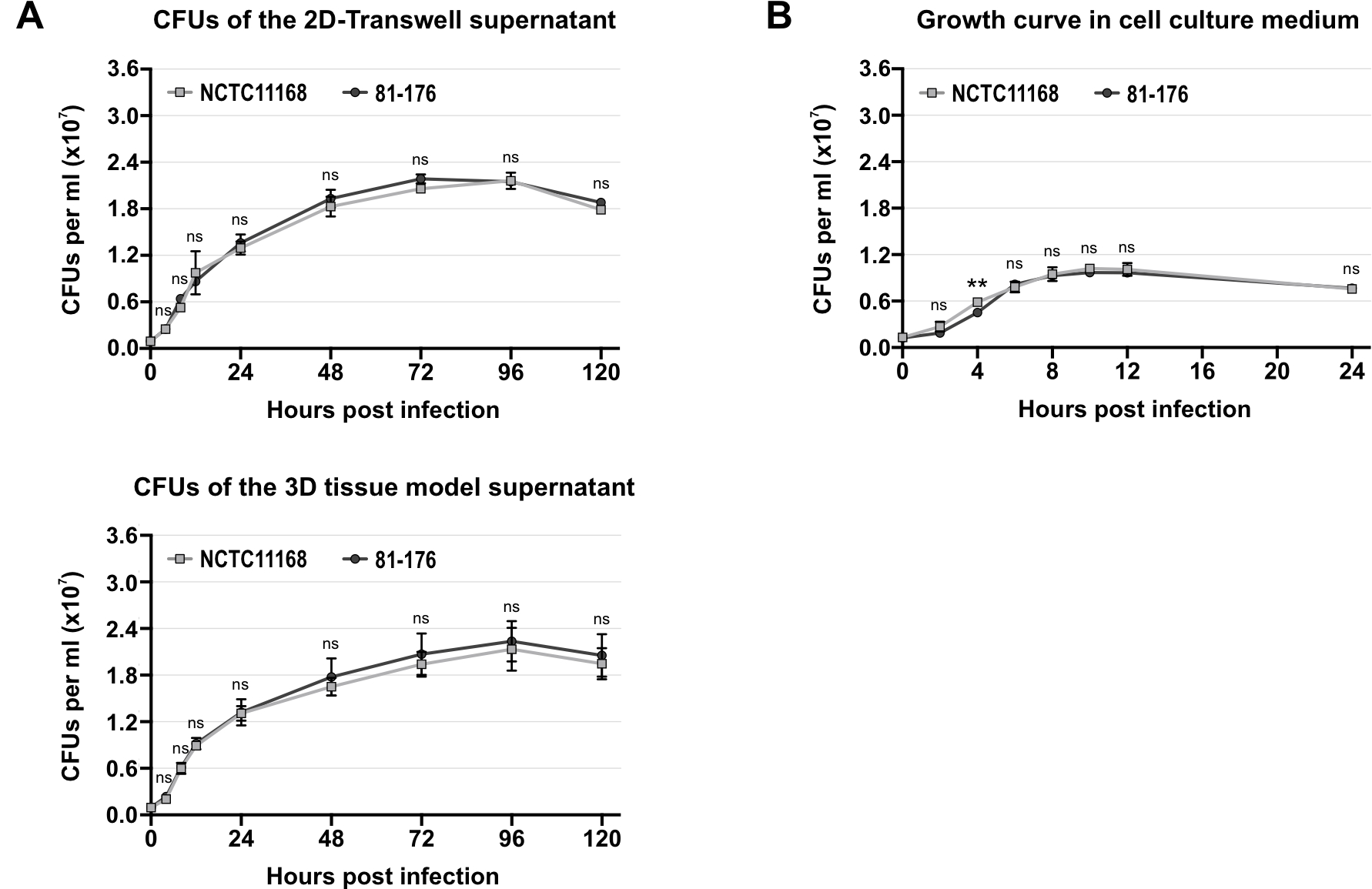

Supplement: S5 Fig — (A, B) Replication of C. jejuni wild-type strains in cell culture medium (MEM + 20% FCS, 1% NEAA, 1% Sodium Pyruvate) supernatant of 2D-Transwells (upper panel) and 3D tissue models (lower panel) during the course of infection (A) or in cell culture medium alone (B). CFUs are depicted as CFUs/ml (x107) and represent the mean of three independent experiments with respective SDs. Statistical significance in (A) and (B) is calculated for the comparison between wild-type strains. **: p < 0.01, ns: not significant, using Student’s t-test. (TIF) [file ppat.1008304.s005.tif]

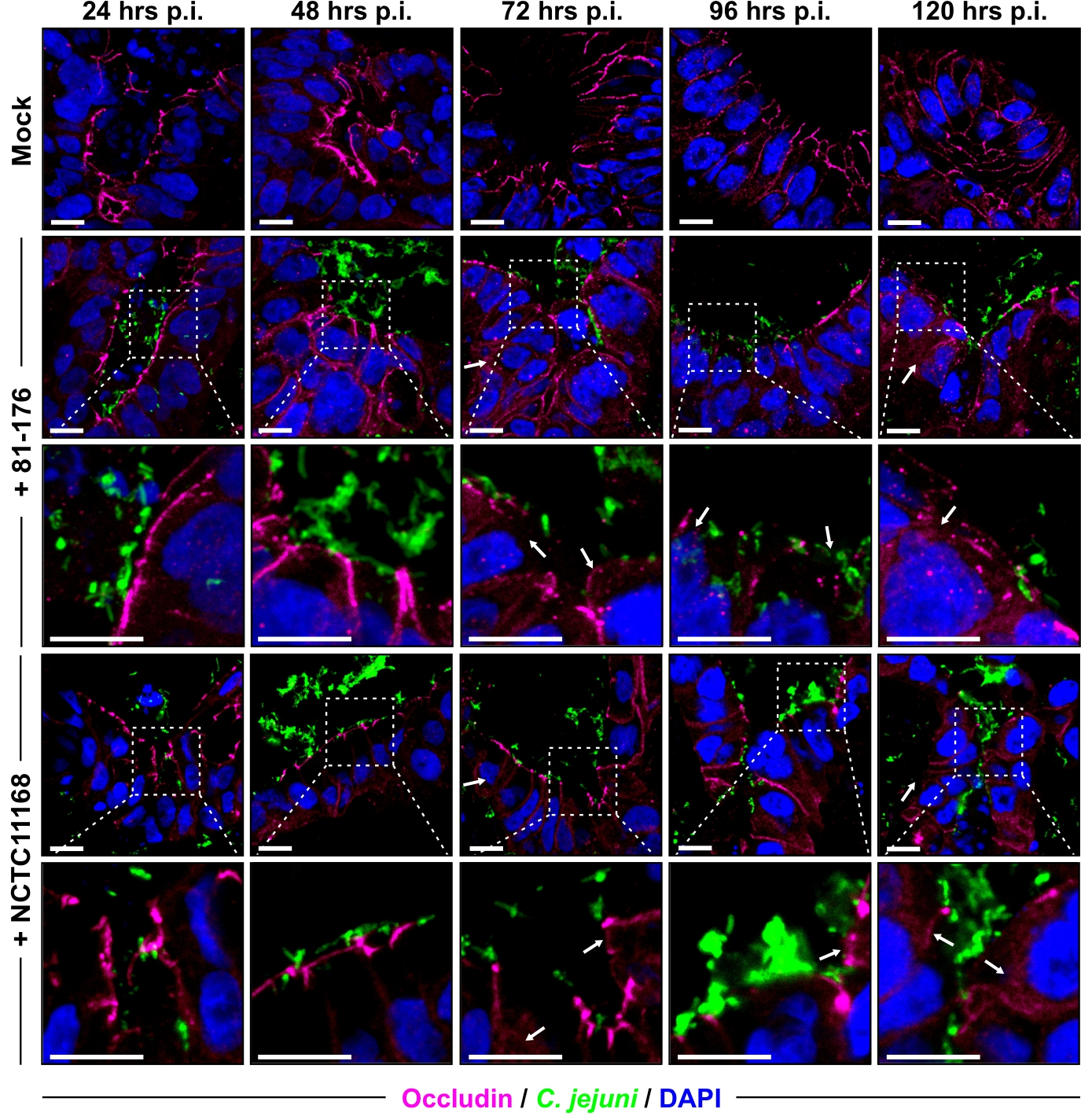

Supplement: S6 Fig — Confocal microscopy images of paraffin sections of the Caco-2 cell-based 3D tissue model cultured dynamically during infection with C. jejuni strains 81–176 and NCTC11168 (24–120 hrs p.i.) or non-infected controls. Bacteria were detected with an anti-C. jejuni antibody (green), nuclei were stained with DAPI (blue), and an anti-occludin antibody was used to visualize TJs (tight junctions, magenta). White arrows indicate regions of redistribution of tight junction staining from the periphery of the cell to intracellular regions as well as loss of apical staining for occludin. Images in the second row for each strain are 3-fold magnifications of the indicated region in the respective confocal image above. Scale bars: 10 μm. (TIF) [file ppat.1008304.s006.tif]

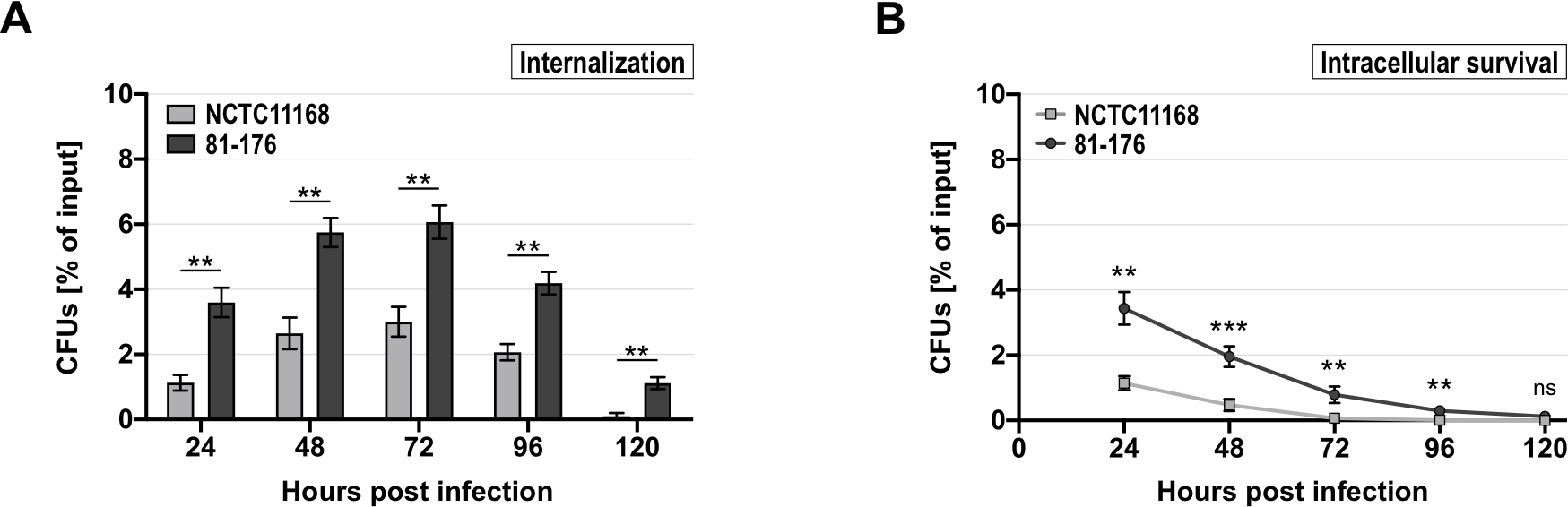

Supplement: S7 Fig — (A) Internalization of C. jejuni NCTC11168 and 81–176 WT strains into the 3D tissue model was determined at each time point after a 2 hrs gentamicin treatment (200 μg/ml) with subsequent isolation of CFUs. Experiments were performed in triplicates and internalized CFUs (percentage of input) are depicted as the mean with corresponding SDs. (B) To determine intracellularly surviving bacteria, 3D tissue models infected with C. jejuni NCTC11168 and 81–176 were treated with 200 μg/ml gentamicin for 2 hrs at the 24 hrs time point only. Subsequently, medium in both apical and basolateral compartments was exchanged for fresh cell culture medium containing 10 μg/ml gentamicin to inhibit growth of bacteria released from host cells. CFUs were recovered at the indicated time points to determine the number of surviving intracellular bacteria and are depicted as the mean of three biological replicates with respective SDs (percentage of input). Statistical significance in both (A) and (B) was calculated for the comparison of CFUs between strains NCTC11168 and 81–176. ***: p < 0.001, **: p < 0.01, ns: not significant, using Student’s t-test. (TIF) [file ppat.1008304.s007.tif]

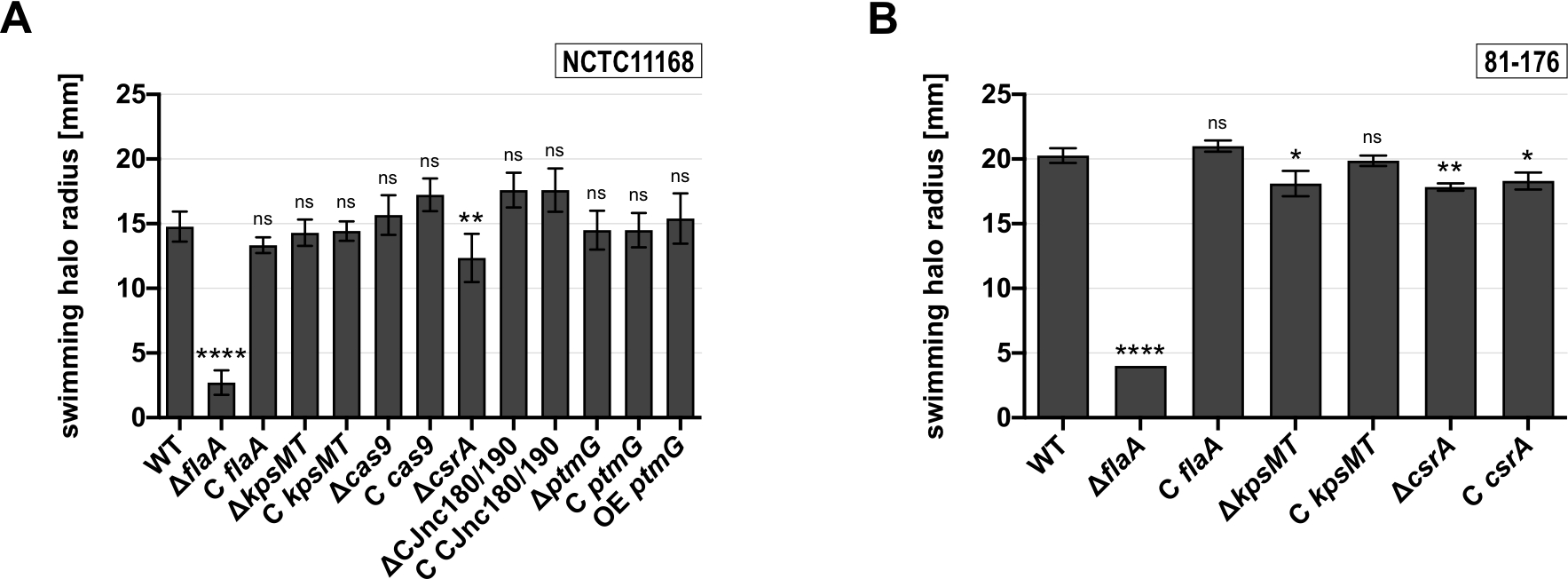

Supplement: S8 Fig — (A) C. jejuni NCTC11168 wildtype, deletion mutants (ΔflaA, ΔkpsMT, Δcas9, ΔcsrA, ΔptmG, ΔCJnc180/190), respective complementation strains (C flaA, C kpsMT, C cas9, C ptmG, C CJnc180/190), and the overexpression mutant OE ptmG were grown overnight in Brucella broth (BB) liquid culture to mid-log phase (OD600 0.4) and subsequently stabbed into 0.4% soft agar BB plates. After 24 hrs of incubation at 37°C in a microaerobic environment, motility was measured by determining the swimming halo radius in comparison to wild-type behavior. (B) C. jejuni 81–176 wildtype, deletion mutants (ΔflaA, ΔkpsMT, ΔcsrA), and respective complementation mutants (C flaA, C kpsMT, C csrA) were assessed for motility as described for NCTC11168 strains above. Bar graphs and corresponding SDs represent the mean of three biological replicates. Statistical significance was calculated for the comparison between each mutant strain and its respective wildtype. ****: p < 0.0001, *: p < 0.05, ns: not significant, using Student’s t-test. (TIF) [file ppat.1008304.s008.tif]

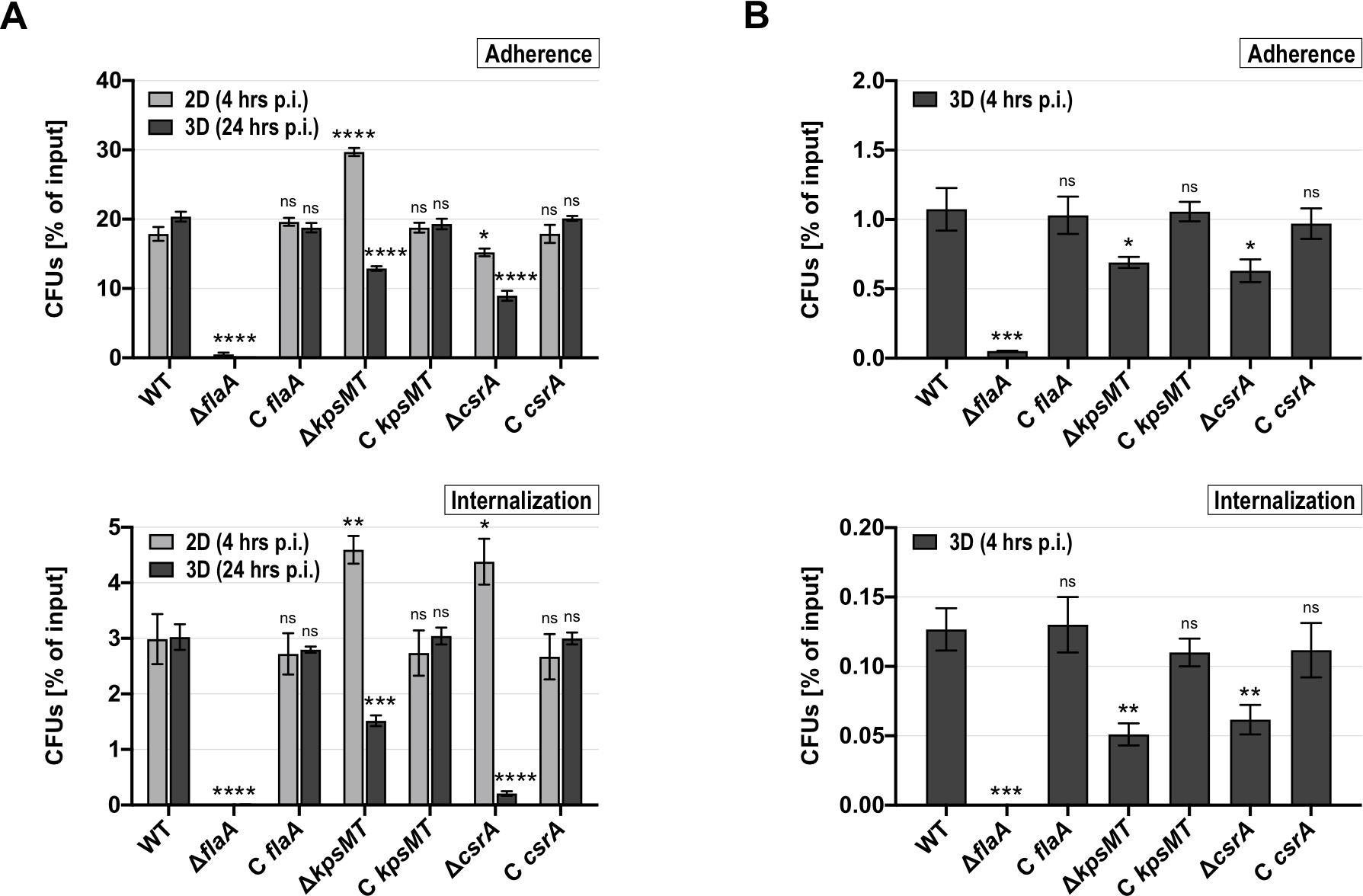

Supplement: S9 Fig — (A, B) Adherence (upper panels) and internalization (lower panels) of C. jejuni 81–176 wildtype (WT), deletion mutants (ΔflaA, ΔkpsMT, ΔcsrA), and their respective complementation strains (C flaA, C kpsMT, C csrA) was examined at 4 hrs p.i. in 2D-monolayers and 24 hrs p.i. in 3D tissue models (A) as well as 4 hrs p.i. in 3D tissue models (B). CFUs are depicted as a percentage of input and represent the mean of three biological replicates with corresponding SDs. Asterisks or ns above each bar indicate the significance of the tested mutant strain compared to their respective wildtype in 2D or 3D. ****: p < 0.0001, ***: p < 0.001, **: p < 0.01, *: p < 0.05, ns: not significant, using Student’s t-test. (TIF) [file ppat.1008304.s009.tif]

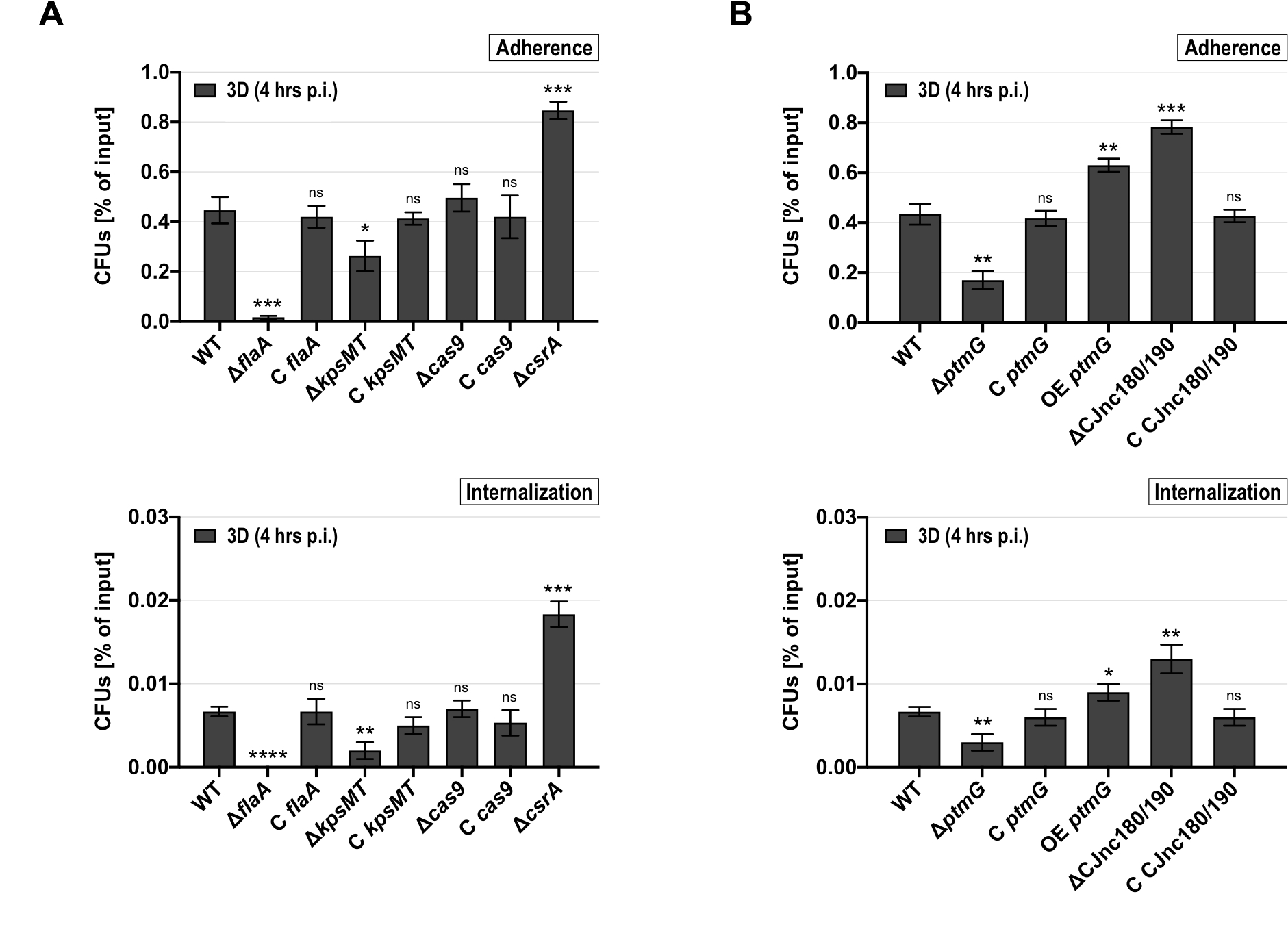

Supplement: S10 Fig — (A, B) Isolation of CFUs from 3D tissue models (4 hrs p.i.) infected with C. jejuni NCTC11168 wildtype (WT) and deletion/complementation mutants from Main Fig 6 for either adherence (upper panels) or internalization (lower panels). CFUs are depicted as the percentage of their respective input CFUs and represent the mean of three biological replicates with corresponding SDs. Asterisks or ns above each bar indicate the significance of the tested mutant strain compared to their respective wildtype in 3D. ****: p < 0.0001, ***: p < 0.001, **: p < 0.01, *: p < 0.05, ns: not significant, using Student’s t-test. (TIF) [file ppat.1008304.s010.tif]
