# Supplementary material for: A three-dimensional intestinal tissue model reveals factors and small regulatory RNAs important for colonization with Campylobacter jejuni
Source: PLoS Pathog. 2020 Feb 18;16(2):e1008304. doi: 10.1371/journal.ppat.1008304 (PMC7048300; doi:10.1371/journal.ppat.1008304)
Supplement: S1 Table — Caco-2 cells of statically cultured 3D tissue models were harvested by trypsin treatment coupled with extensive mechanical dissolution. Subsequently, cells were counted in a Neubauer counting chamber using the trypan blue exclusion method. (DOCX) [file ppat.1008304.s011.docx]

**S1 Table. Cell counting of statically cultured tissue models.** Caco-2 cells of statically cultured 3D tissue models were harvested by trypsin treatment coupled with extensive mechanical dissolution. Subsequently, cells were counted in a Neubauer counting chamber using the trypan blue exclusion method.

| **Tissue model** | **No. of cells** | **Tissue model** | **No. of cells** | **Tissue model** | **No. of cells** |
| --- | --- | --- | --- | --- | --- |
| crown #01 | 620,000 | crown #09 | 650,000 | crown #17 | 590,000 |
| crown #02 | 680,000 | crown #10 | 680,000 | crown #18 | 675,000 |
| crown #03 | 600,000 | crown #11 | 690,000 | crown #19 | 650,000 |
| crown #04 | 675,000 | crown #12 | 550,000 | crown #20 | 700,000 |
| crown #05 | 590,000 | crown #13 | 635,000 | crown #21 | 660,000 |
| crown #06 | 620,000 | crown #14 | 675,000 | crown #22 | 660,000 |
| crown #07 | 675,000 | crown #15 | 640,000 | crown #23 | 650,000 |
| crown #08 | 710,000 | crown #16 | 600,000 | crown #24 | 630,000 |
| **Average number of Caco-2 cells** | | | | | **650,000** |
