# Supplementary material for: A three-dimensional intestinal tissue model reveals factors and small regulatory RNAs important for colonization with Campylobacter jejuni
Source: PLoS Pathog. 2020 Feb 18;16(2):e1008304. doi: 10.1371/journal.ppat.1008304 (PMC7048300; doi:10.1371/journal.ppat.1008304)
Supplement: S2 Table — DNA content of 300,000 and 600,000 Caco-2 cells as well as three statically cultured 3D tissue models was determined using the Quant-iT PicoGreen dsDNA assay kit. The relative fluorescence intensities designated with an asterisk indicate the final intensity after subtraction of background fluorescence of unseeded SISmuc. (DOCX) [file ppat.1008304.s012.docx]

**S2 Table. PicoGreen assay of statically cultured tissue models.** DNA content of 300,000 and 600,000 Caco-2 cells as well as three statically cultured 3D tissue models was determined using the Quant-iT PicoGreen dsDNA assay kit. The relative fluorescence intensities designated with an asterisk indicate the final intensity after subtraction of background fluorescence of unseeded SISmuc.

|  | **Relative fluorescence intensity** | **DNA content [ng/ml]** | **Calculated No. of Caco-2 cells** |
| --- | --- | --- | --- |
| 300,000 Caco-2 cells | 12433.176 | 219 | − |
| 600,000 Caco-2 cells | 23811.072 | 423 | − |
| crown #01 | 33515.748* | 597 | 830,000 |
| crown #02 | 20855.050* | 370 | 520,000 |
| crown #03 | 28551.862* | 508 | 710,000 |
| **Average number of Caco-2 cells** | | | **690,000** |
