# Supplementary material for: A three-dimensional intestinal tissue model reveals factors and small regulatory RNAs important for colonization with Campylobacter jejuni
Source: PLoS Pathog. 2020 Feb 18;16(2):e1008304. doi: 10.1371/journal.ppat.1008304 (PMC7048300; doi:10.1371/journal.ppat.1008304)
Supplement: S3 Table — As for the static tissue models (S1 Table), Caco-2 cells of dynamically cultured 3D tissue models were harvested and counted. (DOCX) [file ppat.1008304.s013.docx]

**S3 Table. Cell counting of dynamically cultured tissue models.** As for the static tissue models (S1 Table), Caco-2 cells of dynamically cultured 3D tissue models were harvested and counted.

| **Tissue model** | **No. of cells** | **Tissue model** | **No. of cells** | **Tissue model** | **No. of cells** |
| --- | --- | --- | --- | --- | --- |
| crown #01 | 675,000 | crown #05 | 685,000 | crown #09 | 630,000 |
| crown #02 | 630,000 | crown #06 | 640,000 | crown #10 | 680,000 |
| crown #03 | 640,000 | crown #07 | 670,000 | crown #11 | 690,000 |
| crown #04 | 690,000 | crown #08 | 690,000 | crown #12 | 720,000 |
| **Average number of Caco-2 cells** | | | | | **670,000** |
