# Supplementary material for: A three-dimensional intestinal tissue model reveals factors and small regulatory RNAs important for colonization with Campylobacter jejuni
Source: PLoS Pathog. 2020 Feb 18;16(2):e1008304. doi: 10.1371/journal.ppat.1008304 (PMC7048300; doi:10.1371/journal.ppat.1008304)
Supplement: S4 Table — (DOCX) [file ppat.1008304.s014.docx]

| **Name** | **Marker** | ***C. jejuni* strain** | **Description** | **Strain** **number /**  **Reference** |
| --- | --- | --- | --- | --- |
| **NCTC11168_WT** |  | NCTC11168 | Wildtype, kindly provided by Arnoud van Vliet (Institute of Food Research, Norwich, UK) | CSS-0032 |
| **NCTC11168_∆*flaA*** | Kan^R^ | NCTC11168 | ∆*flaA*::*aphA-3* | CSS-1512 / [[1]](http://f1000.com/work/citation?ids=4102583&pre=&suf=&sa=0) |
| **C *flaA*** | Kan^R^  Cm^R^ | NCTC11168 | ∆*flaA*::*aphA-3*  *rdxA*::*cat.coli*::*flaA* | CSS-6208/  This study |
| **∆*kpsMT*** | Kan^R^ | NCTC11168 | ∆*kpsMT*::*aphA-3* | CSS-6198 /  This study |
| **C *kpsMT*** | Kan^R^  Cm^R^ | NCTC11168 | ∆*kpsMT*::*aphA-3*  *rdxA*::*cat.coli*::*kpsMT* | CSS-6213 /  This study |
| **∆*cas9*** | Hyg^R^ | NCTC11168 | ∆*cas9*::*aph(7’’)* | CSS-3836 / [[2]](http://f1000.com/work/citation?ids=4914191&pre=&suf=&sa=0) |
| **C *cas9*** | Hyg^R^  Kan^R^ | NCTC11168 | ∆*cas9*::*aph(7’’)aphA-3*  *rdxA*::*cas9*::*aphA-3* | CSS-3858 / [[2]](http://f1000.com/work/citation?ids=4914191&pre=&suf=&sa=0) |
| **∆*csrA*** | Cm^R^ | NCTC11168 | ∆*csrA*::*cat.coli* | CSS-0643 / [[1]](http://f1000.com/work/citation?ids=4102583&pre=&suf=&sa=0) |
| **∆*ptmG*** | Hyg^R^ | NCTC11168 | ∆*ptmG*::*aph(7’’)* | CSS-2966 /  This study |
| **C *ptmG*** | Hyg^R^  Kan^R^ | NCTC11168 | ∆*ptmG*::*aph(7’’),*  *rdxA*::*ptmG*::*aphA-3* | CSS-2978 /  This study |
| **OE *ptmG*** | Kan^R^ | NCTC11168 | *rdxA*::*ptmG*::*aphA-3* | CSS-2980 /  This study |
| **∆CJnc180/190** | Kan^R^ | NCTC11168 | ∆CJnc180/190::*aphA-3* | CSS-1157 /  This study |
| **C CJnc180/190** | Kan^R^  Cm^R^ | NCTC11168 | ∆CJnc180/190::*aphA-3,*  *rdxA*::*cat.coli*::CJnc180/190 | CSS-1158 /  This study |
| **81-176_WT** |  | 81-176 | Wildtype, kindly provided by Patricia Guerry (Naval Medical Research Center, Silver Spring, MD, USA) | CSS-0063 |
| **81-176_∆*flaA*** | Kan^R^ | 81-176 | ∆*flaA*::*aphA-3* | CSS-2380 /  This study |
| **C *flaA*** | Kan^R^  Cm^R^ | 81-176 | ∆*flaA*::*aphA-3* | CSS-6340/  This study |
| **∆*kpsMT*** | Kan^R^ | 81-176 | ∆*kpsMT*::*aphA-3* | CSS-6200 /  This study |
| **C *kpsMT*** | Kan^R^  Cm^R^ | 81-176 | ∆*kpsMT*::*aphA-3*  *rdxA*::*cat.coli*::*kpsMT* | CSS-6215/  This study |
| **∆*csrA*** | Cm^R^ | 81-176 | ∆*csrA*::*cat.coli* | CSS-6202 /  This study |
| **C *csrA*** | Kan^R^  Cm^R^ | 81-176 | ∆*csrA*::*cat.coli*  *rdxA*::*csrA*::*aphA-3* | CSS-6334 /  This study |

**S4 Table. Bacterial strains.**

Kan^R^: kanamycin resistance; Hyg^R^: hygromycin resistance; Cm^R^: chloramphenicol resistance

1. Dugar G, Svensson SL, Bischler T, Wäldchen S, Reinhardt R, Sauer M, *et al.* (2016) The CsrA-FliW network controls polar localization of the dual-function flagellin mRNA in *Campylobacter jejuni*. Nat Commun 7: 11667. doi:10.1038/ncomms11667.

2. Dugar G, Leenay RT, Eisenbart SK, Bischler T, Aul BU, Beisel CL, *et al.* (2018) CRISPR RNA-Dependent Binding and Cleavage of Endogenous RNAs by the *Campylobacter jejuni* Cas9. Mol Cell 69: 893–905.e7. doi:10.1016/j.molcel.2018.01.032.
