# Supplementary material for: A three-dimensional intestinal tissue model reveals factors and small regulatory RNAs important for colonization with Campylobacter jejuni
Source: PLoS Pathog. 2020 Feb 18;16(2):e1008304. doi: 10.1371/journal.ppat.1008304 (PMC7048300; doi:10.1371/journal.ppat.1008304)
Supplement: S5 Table — (DOCX) [file ppat.1008304.s015.docx]

**S5 Table. DNA oligonucleotides.**

| **Name** | **Sequence 5’ → 3’** | **Description** |
| --- | --- | --- |
| **CSO-0023** | CCACCAGCTTATATACCTTAGCA | Validation of C *ptmG* |
| **CSO-0246** | GAAAGGTAAACAATGAAAAGTGATT | Validation of ∆CJnc180/190 |
| **CSO-0247** | GTGAATGAATTTAAAGCAAATCA | Cloning of ∆CJnc180/190 |
| **CSO-0248** | ATTGCACTTATGGGGGTGTTTTTGCTTAAAGAGAACAAACGAT | Cloning of ∆CJnc180/190 |
| **CSO-0249** | GATTTTAAAAATTATCCATAAAATGACATTTTATTCTAAAAACACTTAATCT | Cloning of ∆CJnc180/190 |
| **CSO-0250** | GATTGATGGGTATTATGGGG | Cloning of ∆CJnc180/190 |
| **CSO-0345** | GTTTTTTCTAGATGATAGGTGGCTTTGAAAAAG | Cloning of intermediary plasmid pGD7.1 |
| **CSO-0347** | GTTTTTATCGATCTCTAGCTAGCATACTTTACAGTGC | Sense primer for cloning of *C. jejuni* *rdxA* complementation vector; used for construction of complementation strains |
| **CSO-0348** | GTTTTTCTCGAGAGTAAAAGCCCTAAAAAAGCTG | Cloning of intermediary plasmid pGD7.1 |
| **CSO-0349** | CTGCAAAAGAAGCATTGACA | Validation of C *ptmG* and C 180/190 |
| **CSO-0350** | GTTTTTCATATGCTCGAATTCAGATCCACGTT | Antisense primer for cloning of *C. jejuni* *rdxA* complementation vector |
| **CSO-0354** | GTTTTTATCGATGCGAAACTATGGATAATGCTG | Cloning of complementation plasmid pGD34.7 (C 180/190) |
| **CSO-0355** | GTTTTTCATATGTTGCCGTGATTAATGGG | Cloning of complementation plasmid pGD34.7 (C 180/190) |
| **CSO-0394** | TGCAAGGAATTATCTCCTATACAC | Cloning of ∆*csrA* in 81-176 |
| **CSO-0395** | ATCATAAACAGCTTTAGTTTGGC | Cloning of ∆*csrA* in 81-176 |
| **CSO-0493** | GTTTTTCTGCAGCTCTAGCTAGCATACTTTACAGTGC | Cloning of complementation plasmid pSSv63.1 (C *ptmG*) |
| **CSO-0573** | TTTATTCAGCAAGTCTTGTAATTCA | Antisense primer for *cat.coli* cassette with terminator |
| **CSO-0613** | AACAAATCGGAATTTACGGA | Sense primer for non-polar *cat.coli* cassette |
| **CSO-0614** | GGCACCAATAACTGCCTTAA | Antisense primer for non-polar *cat.coli* cassette |
| **CSO-0615** | CTCCGTAAATTCCGATTTGTTCTTGATAATATTAACATTTTTCAACCT | Cloning of ∆*csrA* in 81-176 |
| **CSO-0616** | TTTTAAGGCAGTTATTGGTGCCGCAAAAAACTAATCAAATGAAAG | Cloning of ∆*csrA* in 81-176 |
| **CSO-0643** | TATTCCCTTATCAATTCAAGTGCATCATGCCG | Validation of C *ptmG* and C CJnc180/190 |
| **CSO-0762** | GTTTTTCCCGGGTCGATACTATGTTATACGCCAA | Cloning of complementation plasmid pSSv63.1 (C *ptmG*) |
| **CSO-1531** | AGCTTGACTATAGAAAGCTTAAATG | Verification of ∆*ptmG* and ∆CJnc180/190 |
| **CSO-1532** | GAAATCATGCAAATTCTTAGAGATAAT | Cloning of ∆*ptmG* |
| **CSO-1535** | GCTTTATCGATGAAATGTATAAAGC | Cloning of ∆*ptmG* |
| **CSO-1536** | TCCTAGTTAGTCACCCGGGTACTCCCCACCCCTTCAATTTAATAA | Cloning of ∆*ptmG* |
| **CSO-1537** | TGTGTTTTAGTACCTGGAGGGAATAAAATATAAAGGAAAAAAATGCAAAACTCAT | Cloning of ∆*ptmG* |
| **CSO-1551** | AGTCGTGTTGTAAGAAATTTGCA | Cloning of ∆*flaA* in 81-176 |
| **CSO-1552** | TCCTAGTTAGTCACCCGGGTATTTAAATCCTTTTAAATAATTTCAAACTC | Cloning of ∆*flaA* in 81-176 |
| **CSO-1553** | ATTGTTTTAGTACCTGGAGGGAATATTAAATCTTAAAATCACTTTACATTCTTT | Cloning of ∆*flaA* in 81-176 |
| **CSO-1554** | TTATAGCTTGACCTAAAGTTGCT | Cloning of ∆*flaA* in 81-176 |
| **CSO-1555** | TATGCAGGCAAAGGTGAAG | Verification of ∆*flaA* in 81-176 |
| **CSO-1678** | GTACCCGGGTGACTAACTAGGGTGACTAACTAGGAGGAATAAATG | Sense primer for *aph(7’’)* |
| **CSO-1679** | TATTCCCTCCAGGTACTAAAACAGTCATATTCCCTCCAGGTATCA | Antisense primer for *aph(7’’)* |
| **CSO-2007** | ACAATATGGATGAAAGAATTTTAGAA | Verification of ∆*kpsMT* |
| **CSO-2008** | TCCTAGTTAGTCACCCGGGTACTCTCTAAAAAATAAAGCATAAATTAC | Cloning of ∆*kpsMT* |
| **CSO-2009** | AGCAGCTTTTATCTTGGGCTA | Cloning of ∆*kpsMT* |
| **CSO-2010** | CCCTAAAAGCAGGATCTCCA | Cloning of ∆*kpsMT* |
| **CSO-2011** | ATTGTTTTAGTACCTGGAGGGAATAGTTAATTAATCTAACAAAATCTTATCC | Cloning of ∆*kpsMT* |
| **CSO-2276** | CTTAATTTAACTTATCCTTTTGAAAC | Cloning of C *ptmG* and C CJnc180/190 |
| **CSO-2277** | CAAGCATTTTATCGGCTAATGG | Cloning of C *ptmG* and C CJnc180/190 |
| **CSO-2928** | GTTTTTCCCGGGCCTTATAAAAAGGAATTTTAGGTAG | Cloning of complementation plasmid pSSv63.1 (C *ptmG*) |
| **CSO-2929** | GTTTTTCTGCAGCTGGAAAAAGTAATCCGTATCC | Cloning of complementation plasmid pSSv63.1 (C *ptmG*) |
| **CSO-3270** | AATATCAAGTGATAAATAAGAATCAAGC | Sequencing of insertions at *rdxA* |
| **CSO-4739** | TCCTAGTTAGTCACCCGGGTACTCCCTAAAAAATAAAGCGTGAAT | Cloning of ∆*kpsMT* in 81-176 |
| **CSO-4740** | ATTGTTTTAGTACCTGGAGGGAATAGCTAATTAATCTAACTAAATCTTATCC | Cloning of ∆*kpsMT* in 81-176 |
| **CSO-4741** | CAAAGTTGGCGTATAACATAGTATCGATCTTTAGAGCGCATTAAAGAAG | Cloning of *csrA* complementation in 81-176 |
| **CSO-4743** | GCACTGTAAAGTATGCTAGCTAGAGATTTGCTTTAGCGTAAGCTTTC | Cloning of *csrA* complementation in 81-176 |
| **CSO-4744** | TGAATTACAAGACTTGCTGAATAAAGAAAAGTTAGAACAAAAATACAAGTC | Cloning of *flaA* complementation in NCTC11168 |
| **CSO-4745** | GCACTGTAAAGTATGCTAGCTAGAGCAAGTTTTAGTTTAATTAAAAATTTTAGAG | Cloning of *flaA* complementation in NCTC11168 |
| **CSO-4746** | TGAATTACAAGACTTGCTGAATAAAGAAATTTTTATTTGCAGATTCCAATTAA | Cloning of *flaA* complementation in 81-176 |
| **CSO-4747** | GCACTGTAAAGTATGCTAGCTAGAGGTTTTAATTTGATTAAAAATTTTAAAG | Cloning of *flaA* complementation in 81-176 |
| **CSO-4748** | TGAATTACAAGACTTGCTGAATAAATTTATGCTTTAGAGCTTTTTTGGC | Cloning of *kpsMT* complementation in NCTC11168 and 81-176 |
| **CSO-4749** | GCACTGTAAAGTATGCTAGCTAGAGCAAACAATTTTAAAAGAATCAAATATAC | Cloning of *kpsMT* complementation in NCTC11168 and 81-176 |
| **HPK1** | GTACCCGGGTGACTAACTAGG | Sense primer for non-polar *aphA-3* |
| **HPK2** | TATTCCCTCCAGGTACTAAAACA | Antisense primer for non-polar *aphA-3* |
| **HPK2-term** | AAACACCCCCATAAGTGCAATTATGGGGATAAATATTCCCTCCAGGTACTAAAACA | Antisense primer for polar *aphA-3* |
| **JVO-5068** | TTTTATGGATAATTTTTAAAATCATTTG | Sense primer for polar *aphA-3* |
