# Supplementary material for: A three-dimensional intestinal tissue model reveals factors and small regulatory RNAs important for colonization with Campylobacter jejuni
Source: PLoS Pathog. 2020 Feb 18;16(2):e1008304. doi: 10.1371/journal.ppat.1008304 (PMC7048300; doi:10.1371/journal.ppat.1008304)
Supplement: S6 Table — (DOCX) [file ppat.1008304.s016.docx]

**S6 Table. Plasmids.**

| **Name** | **Description/Generation** | **Origin/**  **Marker** | **Reference** |
| --- | --- | --- | --- |
| **pAC1H** | **Plasmid carrying hygromycin resistance cassette (*aph(7’’)*)** | ColE1/  pBR332/  Hyg^R^ | [[3]](http://f1000.com/work/citation?ids=4468194&pre=&suf=&sa=0) |
| **pGD7.1** | **Intermediary plasmid for construction of pGD34.7, complementation of CJnc180/190 deletion with CJnc180/190 in *C. jejuni* strain NCTC11168.** Insertion of the *rdxA* (Cj1066) locus of *C. jejuni* strain NCTC11168 into pJV752.1 cloning vector. Ligation of *Xba*I/*Xho*I-digested PCR product amplified with CSO-0345/0348 on gDNA of *C. jejuni* NCTC11168 and pJV752.1 (*Dpn*I/*Xba*I/*Xho*I-digested). | p15Amod/Amp^R^ | G. Dugar,  Sharma lab  This study |
| **pGD34.7** | **Plasmid for complementation of CJnc180/190 deletion with CJnc180/190 in *C. jejuni* strain NCTC11168 using *C. coli cat* cassette.** Ligation of *Nde*I/*Cla*I-digested PCR product amplified with CSO-0354/0355 on gDNA of *C. jejuni* NCTC11168 and *Nde*I/*Cla*I-digested PCR product amplified with CSO-0347/0350 on pGD7.1 (*Dpn*I-digested), into which the *cat.coli* cassette (amplified from *C. jejuni* NCTC11168 ∆*csrA*, CSS-0643) had been inserted. | p15Amod/  Amp^R^, Cm^R^ | G. Dugar,  Sharma lab  This study |
| **pGG1** | **Plasmid carrying kanamycin resistance cassette  (*aphA-3*)** | ColE1/  Kan^R^ | [[1]](http://f1000.com/work/citation?ids=4102583&pre=&suf=&sa=0) |
| **pJV752.1** | **Cloning vector, pZE12-*luc* with modified p15A**  **origin** | p15Amod/  Amp^R^ | [[4]](http://f1000.com/work/citation?ids=4770633&pre=&suf=&sa=0) |
| **pSSv63.1** | **Plasmid for complementation of *ptmG* deletion with *ptmG* in *C. jejuni* strain NCTC11168 using *aphA-3* cassette.** Ligation of *Pst*I/*Xma*I-digested PCR product amplified with CSO-2928/2929 on gDNA of *C. jejuni* NCTC11168 and *Pst*I/*Xma*I-digested PCR product amplified with CSO-0762/0493 on pST1.1 (*Dpn*I-digested). | p15Amod/  Amp^R^, Kan^R^ | S. Svensson,  Sharma lab  This study |
| **pST1.1** | ***Campylobacter* complementation plasmid carrying kanamycin resistance cassette (*aphA-3*)** | p15Amod/  Amp^R^, Kan^R^ | [[2]](http://f1000.com/work/citation?ids=4914191&pre=&suf=&sa=0) |

Amp^R^: ampicillin resistance; Kan^R^: kanamycin resistance; Hyg^R^: hygromycin resistance;

Cm^R^: chloramphenicol resistance

1. Dugar G, Svensson SL, Bischler T, Wäldchen S, Reinhardt R, Sauer M, *et al.* (2016) The CsrA-FliW network controls polar localization of the dual-function flagellin mRNA in *Campylobacter jejuni*. Nat Commun 7: 11667. doi:10.1038/ncomms11667.

2. Dugar G, Leenay RT, Eisenbart SK, Bischler T, Aul BU, Beisel CL, *et al.* (2018) CRISPR RNA-Dependent Binding and Cleavage of Endogenous RNAs by the *Campylobacter jejuni* Cas9. Mol Cell 69: 893–905.e7. doi:10.1016/j.molcel.2018.01.032.

3. Cameron A, Gaynor EC (2014) Hygromycin B and apramycin antibiotic resistance cassettes for use in *Campylobacter jejuni*. PLoS One 9: e95084. doi:10.1371/journal.pone.0095084.

4. Sharma CM, Darfeuille F, Plantinga TH, Vogel J (2007) A small RNA regulates multiple ABC transporter mRNAs by targeting C/A-rich elements inside and upstream of ribosome-binding sites. Genes Dev 21: 2804–2817. doi:10.1101/gad.447207.
